# Crosstalk between 5-methylcytosine and N^6^-methyladenosine machinery defines disease progression, therapeutic response and pharmacogenomic landscape in hepatocellular carcinoma

**DOI:** 10.1186/s12943-022-01706-6

**Published:** 2023-01-10

**Authors:** Yu Tian, Haijuan Xiao, Yanhui Yang, Pingping Zhang, Jiahui Yuan, Wei Zhang, Lijie Chen, Yibao Fan, Jinze Zhang, Huan Cheng, Tingwei Deng, Lin Yang, Weiwei Wang, Guoyong Chen, Peiqin Wang, Peng Gong, Xing Niu, Xianbin Zhang

**Affiliations:** 1 Department of General Surgery and Integrated Chinese and Western Medicine, Institute of Precision Diagnosis and Treatment of Digestive System Tumors, Carson International Cancer Center, Shenzhen University General Hospital, Shenzhen University, Shenzhen, Guangdong 518055 China; 2 Department of Oncology, Shaanxi Province, Affiliated Hospital of the Shaanxi University of Traditional Chinese Medicine, Xianyang, Shaanxi 712046 China; 3 Department of Trauma Surgery, The First Affiliated Hospital and College of Clinical Medicine, Henan University of Science and Technology, Luoyang, 471003 Henan China; 4 Department of Gastroenterology, Changhai Hospital, Naval Medical University, Shanghai, 200433 China; 5 China Medical University, Shenyang, 110122 Liaoning China; 6 Department of Hepatobiliary Surgery, Shaanxi Province, Xianyang Central Hospital, Xianyang, 712099 Shaanxi China; 7 Hepatobiliary Surgery, People’s Hospital of Zhengzhou University and Henan Provincial People’s Hospital, Zhengzhou, 450001 Henan China; 8 Department of Gastroenterology, Changzheng Hospital, Naval Medical University, Shanghai, 200003 China

**Keywords:** Hepatocellular carcinoma, 5-methylcytosine, N^6^-methyladenosine, Therapeutic response, Pharmacogenomic landscape, Multi-omics

## Abstract

**Background:**

Accumulated evidence highlights the significance of the crosstalk between epigenetic and epitranscriptomic mechanisms, notably 5-methylcytosine (5mC) and N^6^-methyladenosine (m^6^A). Herein, we conducted a widespread analysis regarding the crosstalk between 5mC and m^6^A regulators in hepatocellular carcinoma (HCC).

**Methods:**

Pan-cancer genomic analysis of the crosstalk between 5mC and m^6^A regulators was presented at transcriptomic, genomic, epigenetic, and other multi-omics levels. Hub 5mC and m^6^A regulators were summarized to define an epigenetic and epitranscriptomic module eigengene (EME), which reflected both the pre- and post-transcriptional modifications.

**Results:**

5mC and m^6^A regulators interacted with one another at the multi-omic levels across pan-cancer, including HCC. The EME scoring system enabled to greatly optimize risk stratification and accurately predict HCC patients’ clinical outcomes and progression. Additionally, the EME accurately predicted the responses to mainstream therapies (TACE and sorafenib) and immunotherapy as well as hyper-progression. In vitro, 5mC and m^6^A regulators cooperatively weakened apoptosis and facilitated proliferation, DNA damage repair, G2/M arrest, migration, invasion and epithelial-to-mesenchymal transition (EMT) in HCC cells. The EME scoring system was remarkably linked to potential extrinsic and intrinsic immune escape mechanisms, and the high EME might contribute to a reduced copy number gain/loss frequency. Finally, we determined potential therapeutic compounds and druggable targets (TUBB1 and P2RY4) for HCC patients with high EME.

**Conclusions:**

Our findings suggest that HCC may result from a unique synergistic combination of 5mC-epigenetic mechanism mixed with m^6^A-epitranscriptomic mechanism, and their crosstalk defines therapeutic response and pharmacogenomic landscape.

**Supplementary Information:**

The online version contains supplementary material available at 10.1186/s12943-022-01706-6.

## Background

Liver cancer, of which 90% are hepatocellular carcinoma (HCC), is the seventh most frequent cancer globally, with 905,677 new diagnosed cases (4.7%) and 830,180 (8.3%) new death cases globally according to the GLOBOCAN 2020 statistics [1]. Most HCC cases arise in the context of chronic liver disease or cirrhosis, the most common cause of which is non-alcoholic fatty liver disease, alcohol-related liver disease, and hepatitis B virus (HBV)/hepatitis C virus (HCV) infection [2]. HCC management is defined by the Barcelona Clinic Liver Cancer (BCLC) staging system that bases on tumor burden, liver function, physical status, etc. [3]. Hepatectomy, ablation and liver transplantation are recommended for patients at the very early stage or early stage, with transarterial chemoembolization (TACE) for those at the intermediate stage, and systemic therapy for those at the advanced stage with adequate liver function and good performance status [3]. Despite the promising treatment options, prognostic outcomes of HCC are still bleak due to the high risk of recurrence and metastasis, with a 5-year survival rate of no more than 12% for advanced HCC [4]. Sorafenib, a tyrosine kinase inhibitor with angiogenic and proliferative effects, is widely applied in the systemic therapy of advanced HCC. However, many HCC patients are not responsive to sorafenib or become resistance within 6 months. Immuno-oncology has become a paradigm shift for the treatment of human cancers, including HCC. The IMbrave150 clinical trial showed that PD-L1 inhibitor (atezolizumab) in combination with VEGF inhibitor (bevacizumab) presented the superior progression-free survival (PFS) and overall survival (OS) in comparison to sorafenib among unresectable HCC patients [5]. Although the combined immunotherapy has been approved as the front-line therapeutic option for advanced HCC patients, the response rate remains ~ 30%. Due to the wide heterogeneity of risk factors and pathogenesis of HCC, established strategies for prediction and prognostication remain limited [6]. Hence, it is critical to ascertain new methods to improve the early diagnosis of HCC and to predict treatment response and survival in patients with established HCC.

Cellular DNA and RNA undergo various forms of methylation. Methylation of DNA and RNA, especially 5-methylcytosine (5mC) and N^6^-methyladenosine (m^6^A) modifications exert key roles in a variety of biological processes [7]. DNA methylation is a well-known and critical epigenetic modification. Studies have revealed that 5mC is the most common type of DNA modification in eukaryotes [8]. m^6^A is the most abundant form of mRNA modification in eukaryotes, which regulates several processes of mRNA metabolism, especially mRNA translation and degradation [9]. Accordingly, several 5mC and m^6^A machines have been well identified. Based on the diverse functions of 5mC and m^6^A machines, 5mC and m^6^A modifications have been shown to impact many fundamental biological processes, including HCC [10, 11]. Rapidly accumulating evidence demonstrates the significant crosstalk between RNA methylation and DNA epigenetic mechanisms in plants [12]. However, there is a lack of knowledge regarding the crosstalk between 5mC and m^6^A regulators in HCC. Thus, the present work aimed to determine the crosstalk of 5mC and m^6^A in HCC, and develop an epigenetic and epitranscriptomic module eigengene (EME) reflecting 5mC and m^6^A modification levels that enabled to define clinical outcomes, therapeutic response and pharmacogenomic landscape.

## Materials and methods:

### Patient cohort for multi-omics profiles

**Fig. 1:**
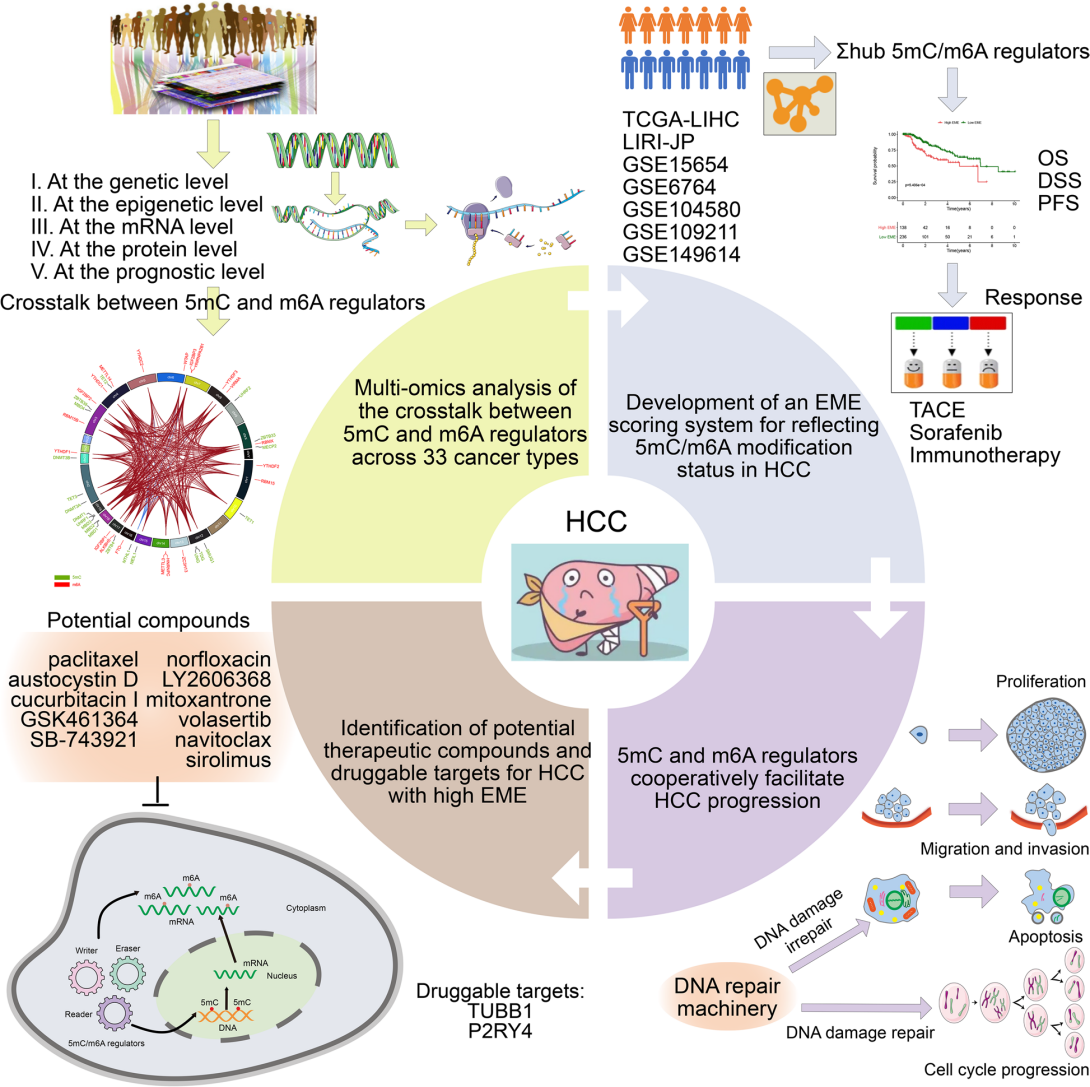
The overall workflow of this study

The overall design of our study is illustrated in Fig. 1. Normalized RNA-seq data on the basis of the Illumina HiSeq platform were obtained for pan-cancer types from the Cancer Genome Atlas (TCGA) through the Xena browser (https://xenabrowser.net/). Raw RNA-seq data were quantified with the root square error method (RSEM), and log2 transformed (RSEM + 1). We retrospectively enrolled independent HCC cohorts from TCGA (https://portal.gdc.cancer.gov/), Gene Expression Omnibus (GEO; https://www.ncbi.nlm.nih.gov/gds/) and ICGC (https://dcc.icgc.org/) databases, including TCGA-LIHC, GSE149614 [13], GSE6764 [14], GSE15654 [15], GSE104580 and LIRI-JP. Raw read counts from TCGA-LIHC were converted to transcripts per kilobase million (TPM) values that were more comparable between samples based on the GENCODE annotation (version 23; https://www.gencodegenes.org/). Microarray data from the Affymetrix® platform were pre-processed through robust multiarray averaging (RMA) algorithm from affy package [16]. Gene expression profiles of LIRI-JP cohort comprised 212 Japanese HCC patients primarily infected with HBV/HCV from the Illumina platform. Raw counts were also converted to TPM values. This study collected a total of 21 5mC and 20 m^6^A regulators from previously published literature. TCGA somatic variants in Mutation Annotation Format (MAF) were also collected and visualized with maftools package [17]. GISTIC 2.0 software was used to identify genes that exhibited significant amplification or deletion [18]. The genomic alterations were quantified through calculating the fractions of genome alteration/gained/lost (FGA/FGG/FGL), defined as the ratio of total CNV/amplification/deletion bases to all bases, respectively. Immune subtypes (C1, wound healing; C2, interferon gamma (IFN-γ) dominant; C3, inflammatory; C4, lymphocyte depleted; C5, immunologically quiet; and C6, transforming growth factor beta (TGF-β) dominant) [19], aneuploidy score, cancer testis antigen (CTA), homologous recombination deficiency (HRD), and intratumor heterogeneity were collected from TCGA dataset or the studies based on TCGA dataset.

The differential expression of 5mC/m^6^A regulators in tumors versus paired normal tissues; heterozygous and homozygous copy number variation (CNV), Pearson correlation between gene expression and CNV; the differential methylation in tumors versus paired normal tissues, the associations of methylation and expression, and prognosis influenced by methylation; spearman correlation analysis of the expression of 5mC/m^6^A regulators with pathway activity (activation or inhibition); and small molecule/drug sensitivity (half-maximal inhibitory concentration (IC50)) were analyzed via the Gene Set Cancer Analysis (GSCALite) web server based on the multi-omics data from 11160 samples across 33 TCGA cancer types, 746 drug sensitivity data from the Genomics of Drug Sensitivity in Cancer (GDSC) and the Cancer Therapeutics Response Portal (CTRP), and normal tissue expression data of 11688 samples from the GTEx [20].

### Development of an epigenetic/epitranscriptomic module eigengene (EME) scoring system

5mC/m^6^A regulators were uploaded to the STRING online database (https://string-db.org/) [21], and a protein–protein interaction (PPI) network was exported. To select hub 5mC/m^6^A regulators, the 41 5mC/m^6^A regulators were used as a module utilizing weighted gene co-expression network analysis (WGCNA) [22]. The summary expression level of the module was defined as the module eigengene via moduleEigengenes function. Then, the module membership (i.e., module eigengene-based intramodular connectivity) was computed as the association between the expression level of a given 5mC/m^6^A regulator and the module eigengene. Hub 5mC/m^6^A regulators were selected as those that had a module membership > 0.7. The overall expression value of the hub 5mC/m^6^A regulators was computed as the EME score. In accordance with the optimal cutoff of EME score, the patients were divided into low or high EME score group.

### Functional enrichment analysis

Gene Ontology (GO) and Kyoto Encyclopedia of Genes and Genomes (KEGG) of hub 5mC/m^6^A regulators were analyzed utilizing clusterProfiler package [23]. The hallmark gene sets were acquired from the Molecular Signatures Database (http://software.broad-institute.org/gsea/msigdb) [24]. Gene set variation analysis (GSVA) was adopted to ascribe signaling pathway variation scores to the gene sets, thus assessing the biological significance [25]. Gene Set Enrichment Analysis (GSEA) was also used to determine whether a defined gene set showed statistically significant between two groups [26].

### Establishment of a nomogram

Independent risk factors derived from multivariate Cox regression analysis, were selected to establish a nomogram for predicting the likelihood of OS via rms package (version 6.2–0). Calibration curves were drawn to assess calibrating capacity. Area under the time‐dependent receiver operating characteristic (ROC) curves (time‐dependent area under the curve (AUC)) and concordance index (C-index) were computed to assess discriminative capacity. The clinical benefits and utility of the nomogram were observed at different threshold probabilities by implementing decision curve analysis.

### Estimation of tumor-infiltrating immune cells

To ensure the reproducibility of the findings, six algorithms were utilized to estimate immune cell infiltrations, comprising single-sample gene set enrichment analysis (ssGSEA), TIMER [27], quanTIseq [28], MCP-counter [29], CIBERSORT [30], and CIBERSORT-ABS [30].

### Therapeutic response analysis

Immunotherapy response was predicted via Immune Cell Abundance Identifier (ImmuCellAI) algorithm [31]. Jiang et al. proposed a signature of T cell dysfunction and exclusion (TIDE) that may accurately estimate immunotherapy response [32]. The response to sorafenib (IC50) was predicted using pRRophetic package based on gene expression levels [33]. RNA-seq and clinical information of 58 patients who received anti-CTLA4 or anti-PD1 immunotherapy were collected from the GSE91061 cohort [34]. The predictive efficacy of the EME was verified in the cohort.

### Cell culture, and RNA interference

Human normal hepatocytes (L-02), and two human primary HCC cell lines (HepG2/Huh-7) were cultured in Dulbecco’s modified Eagle’s medium (DMEM; SH30243.01B; Hyclone, USA) supplemented with 10% fetal bovine serum (FBS; SH30084.03; Hyclone), 100 mg/mL streptomycin, and 100 unit/mL penicillin at 37 °C in 5% CO_2_. Cells at 80% confluence were transfected with siRNA oligonucleotides via Lipofectamine 3000 (Invitrogen, USA). After 24-h incubation, they were harvested for subsequent analyses.

### Protein extraction and western blotting

Protein lysates of cells were extracted with RIPA lysis buffer (BL504A; Biosharp, China). The concentration of the extracted protein was examined with bicinchoninic acid kit (BL521A; Biosharp). Afterwards, equal volume of protein was separated via 8 ~ 12% sodium dodecyl sulfate (SDS)-PAGE gels (SW109-01; SEVEN Biotech, China) and transferred onto polyvinylidene fluoride membranes. The membranes were blocked in blocking buffer and incubated with primary antibody against DNMT1 (1:500; 24206–1-AP; Proteintech, China), METTL3 (1:500; 15073–1-AP; Proteintech), TET3 (1:500; ab139311; Abcam, USA), YTHDC1 (1:500; ab122340; Abcam), TUBB1 (1:1000; ab108342), P2RY4 (1:500; ab180718) or GAPDH (1:5000; 10494–1-AP; Proteintech) overnight at 4 °C. Thereafter, the membranes were incubated with horseradish peroxidase (HRP)-conjugated secondary antibody (1:5000; SA00001-2; Proteintech) for 1 h at room temperature. The development of protein bands was implemented with enhanced chemiluminescence method (BMU101-CN; Abbkine, China), and grey value was quantified utilizing Image J (National Institutes of Health, USA), with GAPDH as an internal reference.

### Cell proliferation detection

For colony formation assay, cells were seeded in 6-well plates (4 × 10^3^ cells / well) for two weeks. After fixation, colonies were dyed utilizing 0.1% crystal violet solution (21155722; Biosharp). 5-Ethynyl-2′-deoxyuridine (EdU) staining was conducted with BeyoClick™ EdU-594 cell proliferation assay kit (C0078S; Beyotime, China) following the manufacturer’s instructions. The percentage of EdU-positive cells was calculated.

### Terminal-deoxynucleoitidyl transferase mediated nick end labeling (TUNEL) staining

TUNEL staining was carried out utilizing TUNEL apoptosis kit (KTA2011; Abbkine) following the manufacturer’s specifications. The percentage of TUNEL-positive cells was also calculated.

### Flow cytometry for cell cycle analysis

Cells were seeded in six-well plates (1 × 10^5^ cells / well). After 24 h, the cells were harvested after trypsinization and washed with ice-cold PBS. The cells were re-suspended in 300 μL PBS plus 5% bovine serum albumin (BSA; V900933; Sigma, USA) and fixed with 700 μL ethanol at 4 °C for 24 h after removing the supernatant following centrifugation. Next, the cells were washed with PBS and centrifuged to discard the ethanol, and re-suspended in 100 μL PBS plus 1 μL ribonuclease A (10 mg/μL). After incubation for half an hour, the cells were stained with 50 μL propidium iodide at 37 °C for half an hour. Cell cycle distribution was evaluated with BD Biosciences FACSCalibur flow cytometry system and analyzed with FlowJo software (BD Biosciences, USA).

### Wound healing

5 × 10^4^ cells were planted onto 6-well plates. When the confluence was up to 90%, the cell monolayer was scratched with a 200 μL pipette tip. After removing detached cells, the remaining cells were cultivated in medium without FBS. Images were investigated at 0, and 24 h and then imaged. Cellular migration was quantified utilizing Image J software.

### Transwell assays

For migration assay, 5 × 10^4^ cells were suspended in 100 μL serum-free DMEM and planted onto the upper chamber of Transwell (8.0 μm) inserts. Meanwhile, 700 μL DMEM supplemented with 10% FBS was added to the lower chamber in a 24-well plate. After 48-h incubation at 37 °C, the migrated cells were fixed by 4% paraformaldehyde (E672002; Sangon Biotech, China) and dyed with 0.1% crystal violet solution for 15 min. For invasion assay, chambers were uniformly covered with 60 μL Matrigel (354,234; BD Biosciences) diluted with DMEM (1:8). After 2-h incubation at 37 °C, cell suspension was planted onto the upper chamber, with 700 μL DMEM supplemented with 10% FBS adding to the lower chamber. Finally, the invasive cells were fixed and dyed.

### Immunofluorescence

After 4% paraformaldehyde fixation, 0.1% Triton X-100 permeabilization, and 3% BSA blockade, HCC cells were incubated with primary antibody against β-catenin (1:100; 51,067–2-AP; Proteintech), E-cadherin (1:100; 20,874–1-AP; Proteintech), or PD-L1 (1:100; 66,248–1-Ig; Proteintech) at 4 °C overnight, followed by incubation with Alexa Fluor-conjugated secondary antibody. Nuclei were counterstained via DAPI (D9542; Sigma). Images were photographed under a fluorescence microscopy (Olympus, Japan) and analyzed with ImageJ software.

### Single-cell sequencing (scRNA-seq) data

The scRNA-seq count matrix of 21 HCC samples was downloaded from the GSE149614 cohort [13]. Quality control was implemented with Seurat package [35]. Single cells with > 20% mitochondrial UMI counts as low-quality cells were removed. Batch effects were eliminated utilizing Integrate Data function. The top 15 principal components (PCs), and the top 1,500 highly variable genes, were selected. Influence of the percentage of mitochondrial UMI counts were removed with ScaleData function. Afterwards, cell populations were clustered with FindClusters function, and visualized with t-distributed stochastic neighbor embedding (t-SNE). The markers of each cell cluster were determined with FindAllMarkers function. The main cell types were determined on the basis of markers acquired from the CellMarker database (http://biocc.hrbmu.edu.cn/CellMarker/ or http://bio-bigdata.hrbmu.edu.cn/CellMarker/) [36].

### Cancer cell line data

We gathered drug sensitivity data of human cancer cell lines (CCLs) from the CTRP (https://portals.broadinstitute.org/ctrp) and PRISM (https://depmap.org/portal/prism/) datasets that were acquired from the Cancer Cell Line Encyclopedia (CCLE) project (https://portals.broadinstitute.org/ccle/). Transcriptome data of the CCLE were employed for CTRP and PRISM analyses. The CERES score that can measure the dependency of genes in specific CCLs were downloaded from the dependency map (DepMap) portal (https://depmap.org/portal/), which was utilized for measuring the gene dependency in specific CCLs. Potential working mechanisms of the identified drugs were analyzed based on the Connectivity Map (CMap) database (https://clue.io) [37].

### Statistical analysis

Data processing, analysis, visualization, etc. were conducted with R packages (version 3.6.3; https://www.bioconductor.org/) or Graphpad Prism software (version 8.0.1; https://www.graphpad.com/). Differences between two groups were evaluated with student’s t or Mann–Whitney U test. One-way analysis of variance (ANOVA) or Kruskal–Wallis test was conducted for multiple comparisons. Fisher's exact test was adopted for analyzing categorical data. Correlation between variables was determined through Pearson or Spearman test. Survival analysis was implemented by Kaplan–Meier approach and log-rank test through survival and survminer packages. Uni- and multivariable Cox regression models were built to calculate hazard ratios (HRs) and identify independent prognostic parameters. ROC curves were plotted using survivalROC package. Principal component analysis (PCA) was utilized to assess the classification accuracy. *P*-value < 0.05 indicated statistical significance.

## Results

### Multi-omics analysis of the crosstalk between 5mC and m^6^A regulators across pan-cancer

This study collected 21 and 20 genes that function as regulators of 5mC-epigenetic and m^6^A-epitranscriptomic mechanisms. The genome-wide omics data of the two regulator classes were analyzed in 33 cancer types. Firstly, we summarized the levels of 5mC and m^6^A regulators across pan-cancer. Most regulators exhibited the co-occurring genetic expression levels in each cancer type (Supplementary Fig. 1A). Additionally, the expression levels of the two regulator classes were comparable across 33 cancer types. Through the GSCALite web server, we investigated the genetic alterations of 5mC and m^6^A regulators across pan-cancer. 5mC and m^6^A regulators presented comparable genetic alterations across 33 cancer types, and the remarkable co-occurrence of genetic alterations was found between the two regulator classes (Supplementary Fig. 1B-E). We also investigated the DNA methylation features of 5mC and m^6^A regulators. Across most cancer types, there was a negative correlation between the mRNA expression and methylation for the same regulator (Supplementary Fig. 2A, B). Interestingly, the two regulator classes exhibited the comparable methylation levels across different cancer types, and their differential methylation status was correlated to survival outcomes (Supplementary Fig. 2C-F). Genomic aberrations affect therapeutic responses and can be potentially applied for drug screening. We observed the significant correlations between drug sensitivity and mRNA expression of most 5mC and m^6^A regulators (Supplementary Fig. 3A, B).

### Landscape of the crosstalk between 5mC and m^6^A regulators in HCC

**Fig. 2:**
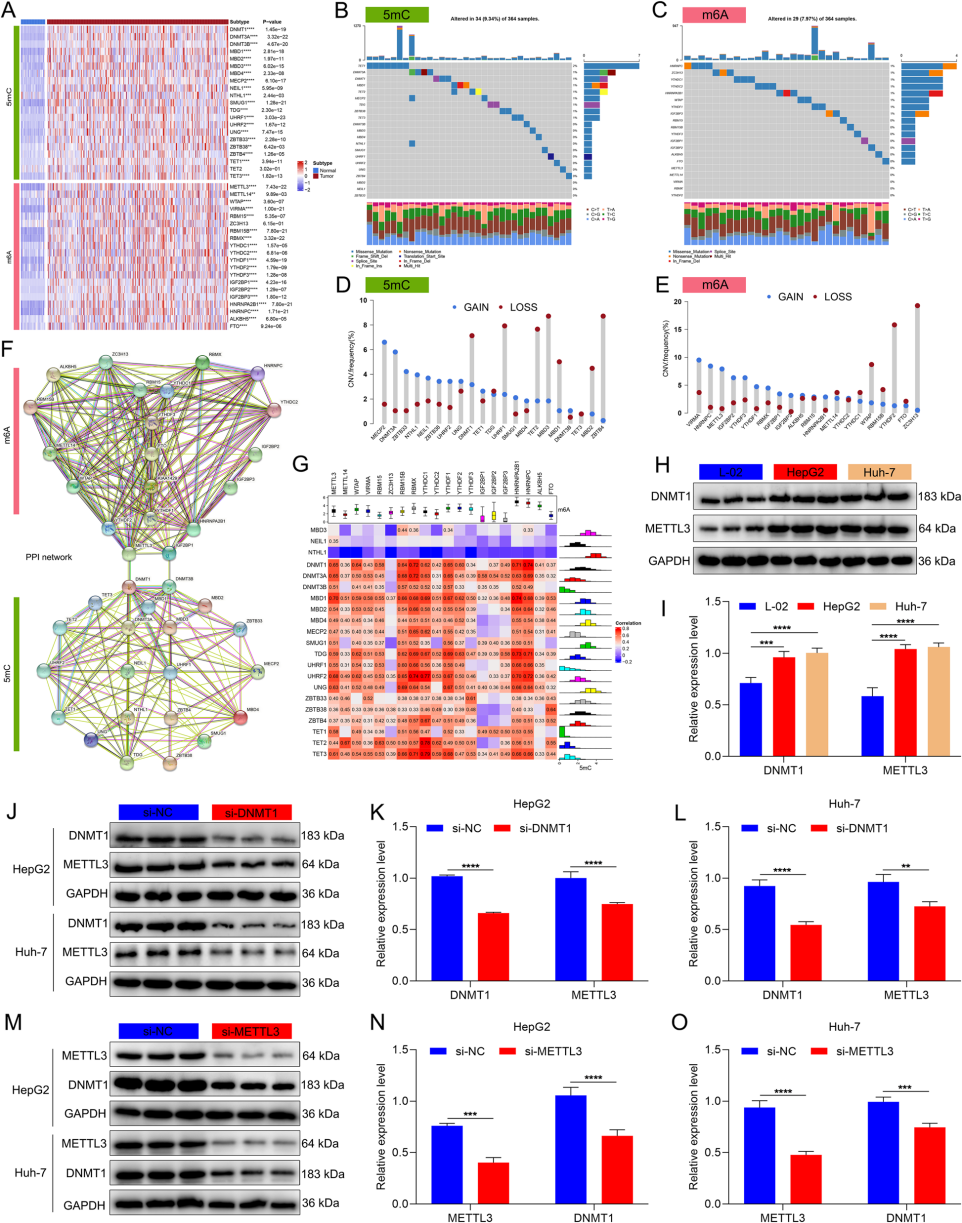
Landscape of the crosstalk between 5mC and m^6^A regulators in HCC. **A** Heatmap showing the differential expression profiles of 5mC/m^6^A regulators in TCGA HCC and normal tissues. **B**, **C** Mutations of 5mC/m^6^A regulators on the basis of TCGA-LIHC cohort. Each column represents each sample, and each row represents each 5mC/m^6^A regulator. Mutation type is marked by unique color. **D**, **E** The CNV features of 5mC/m^6^A regulators in the TCGA-LIHC cohort. The frequencies of copy number gain (blue) and loss (red) are shown. **F** The PPI network of m^6^A (upper) and 5mC (lower) regulators. **G** Correlations between 5mC and m^6^A regulators at the transcriptomic levels in HCC. **H**, **I** Western blot for the expression of DNMT1 and METTL3 in L-02, HepG2 and Huh-7 cells. **J**-**O** Western blot for the expression of DNMT1 and METTL3 in the presence or absence of (**J**-**L**) si-DNMT1 or (**M–O**) si-METTL3 in HepG2 and Huh-7 cells. **p-value < 0.01; ***p-value < 0.001; ****p-value < 0.0001

We further evaluated the differential expression profiling of 5mC and m^6^A regulators in HCC using available tumor and normal tissue expression data. Almost all 5mC and m^6^A regulators were remarkably up-regulated in HCC than normal tissues (Fig. 2A), which reflected the critical significance of epigenetic and epitranscriptomic regulation mechanisms in HCC. As illustrated in Fig. 2B and C, genetic mutations of 5mC and m^6^A regulators were not frequent in HCC. As for CNV characteristics, a majority of 5mC and m^6^A regulators displayed prevalent copy number amplifications or deletions (Fig. 2D, E). The above analysis revealed the transcriptomic and genomic characteristics of 5mC and m^6^A regulators in HCC. Further investigation showed that the 5mC and m^6^A regulators interacted with one another frequently (Fig. 2F). Moreover, at the transcriptomic levels, positive interactions between 5mC and m^6^A regulators were observed (Fig. 2G). For example, DNMT1 and METTL3 were both highly expressed in two HCC cell lines (HepG2 and Huh-7) than normal hepatocytes L-02 (Fig. 2H, I). Silencing DNMT1 or METTL3 significantly attenuated the expression of the other in HepG2 and Huh-7 cell lines (Fig. 2J-O). Similarly, it was proven that the expression of TET3 and YTHDC1 influenced each other in HCC cells (Supplementary Fig. 4A-F).

### Development of an EME scoring system for reflecting 5mC/m^6^A modification status in HCC

**Fig. 3:**
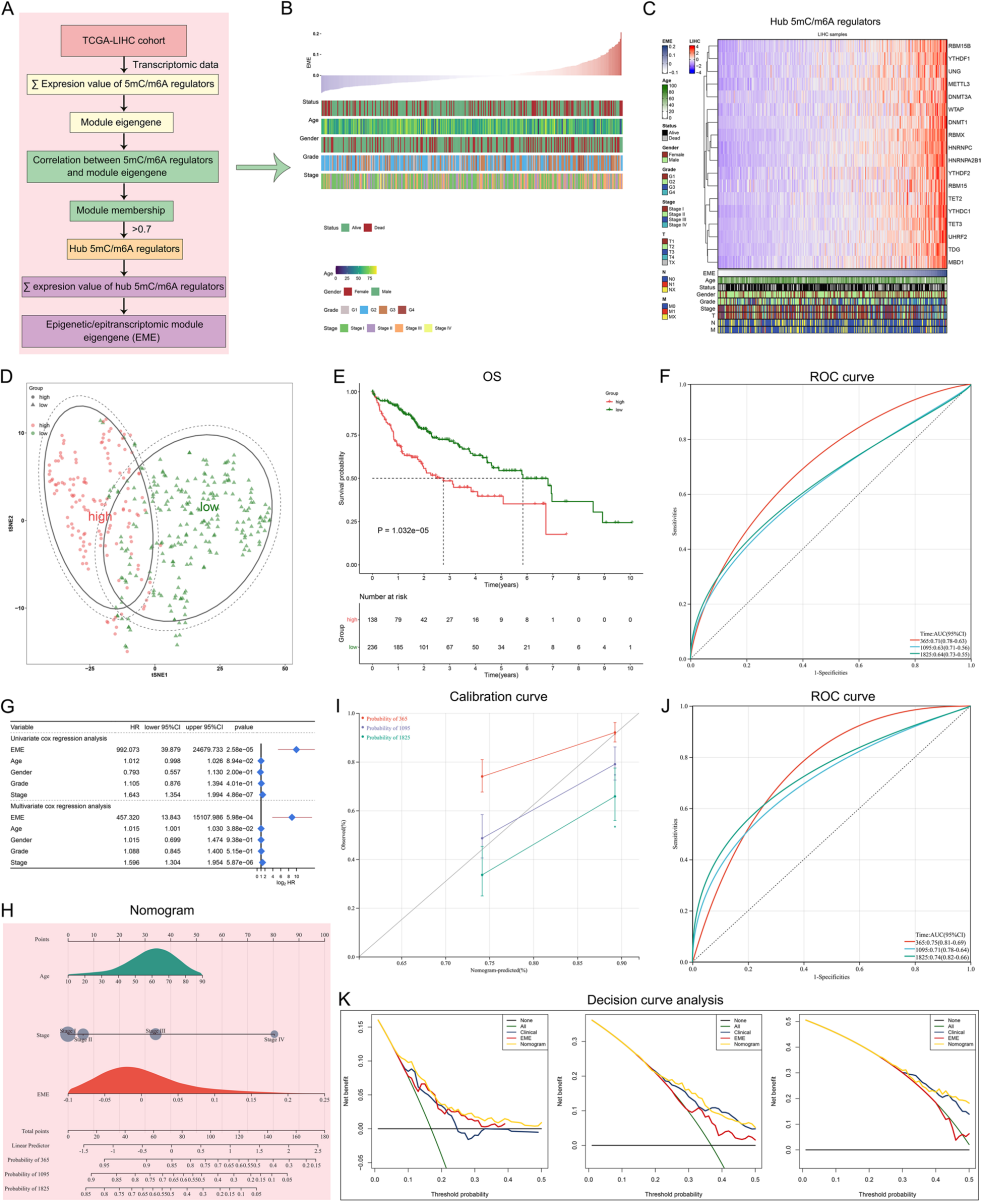
Development of an EME scoring system for reflecting 5mC/m^6^A modification status and predicting clinical outcomes in HCC. **A** Overview of the hub 5mC/m^6^A regulators and the EME scoring system. **B** The distribution of EME and clinical parameters across HCC patients. **C** The expression of the hub 5mC/m^6^A regulators along the EME. **D** PCA for the dissimilarity between the high and low EME subsets according to the expression matrix of the hub 5mC/m^6^A regulators. **E** Kaplan–Meier OS analysis for subgroup patients stratified by the EME. **F** ROCs of the EME for risk prediction of 1-, 3- and 5-year OS. **G** Univariate and multivariate Cox regression analysis of the EME and clinicopathological parameters. **H** Generation of a nomogram combining the EME with other independent clinicopathological factors. **I** Calibration curves for comparison of the nomogram-predicted and actual survival probabilities. The x-axis denotes the nomogram-predicted survival probability, and the y-axis denotes the actual survival probability. The diagonal line indicates the perfect prediction by an ideal model. **J** ROC curves of the nomogram for risk prediction. **K** Decision curve analysis of survival benefits

Pan-cancer survival analysis showed that 5mC and m^6^A regulators played similar roles in OS outcomes of pan-cancer (Supplementary Fig. 5A). Most regulators served as risk factors of HCC patients’ OS (Supplementary Fig. 5B). To determine the hub regulators involved in 5mC and m^6^A modifications in HCC, we employed WGCNA to select hub 5mC/m^6^A regulators that exhibited the remarkably high correlations, which can be explained by their crosstalk. Functional enrichment analysis proved the key roles of hub 5mC/m^6^A regulators in epigenetic and epitranscriptomic processes (Supplementary Fig. 6A-D). An EME scoring system was developed to reflect both the 5mC-epigenetic and m^6^A-epitranscriptomic modification levels through calculating the overall expression levels of hub 5mC/m^6^A regulators (Fig. 3A). According to the optimal cutoff value (0.004) of EMEs, TCGA-LIHC cohort was separated into the high- and low-EME subsets (Fig. 3B, C). The t-SNE highlighted the distinct transcriptional program for two subsets defined by hub 5mC/m^6^A regulators (Fig. 3D).

### The EME scoring system for risk stratification and OS outcomes of individual HCC patients

The clinical outcomes of high- and low-EME HCC patients remarkably varied. Patient with high EME had shorter OS relative to those with low EME (Fig. 3E). To quantify the capacity of this scoring system to predict OS, ROC curves were generated. The 1-, 3-, and 5-year AUC values were 0.71, 0.63 and 0.64, respectively, implying that the scoring system performed well and had the excellent predictive ability (Fig. 3F). To examine the independence of the EME, uni- and multivariate cox regression analyses were implemented. After adjusting by common clinical parameters, the EME still exhibited a robust assessment capacity in OS (Fig. 3G). To further confirm the robustness and superiority of the EME, we implemented subgroup analysis according to age, gender, grade, stage, T stage, and HBV infection. In all subgroups, patients with high EME presented shorter OS than those with low EME (Supplementary Fig. 7A-L). Overall, the EME could greatly optimize risk stratification as well as accurately predict HCC prognosis.

To quantify the risk evaluation for individual HCC patients, a personalized scoring nomogram that combined the EME with clinicopathological parameters (age and stage) was generated for predicting 1-, 3- and 5-year OS probability (Fig. 3H). Based upon the calibration curves, the nomogram exhibited the excellent performance in comparison to an ideal model (Fig. 3I). ROC curves also showed that the nomogram had the strong clinical significance for predicting OS (Fig. 3J). Moreover, the C-index was 0.668. In summary, the nomogram for OS had a considerable discriminative and calibrating capacity. The clinical benefits of the nomogram were also evaluated by decision curve analysis. In Fig. 3K, the nomogram enabled to better predict 1-, 3-, and 5-year OS, because it added more net benefits for almost all threshold probabilities.

### The excellent performance of the EME scoring system in predicting HCC progression

**Fig. 4:**
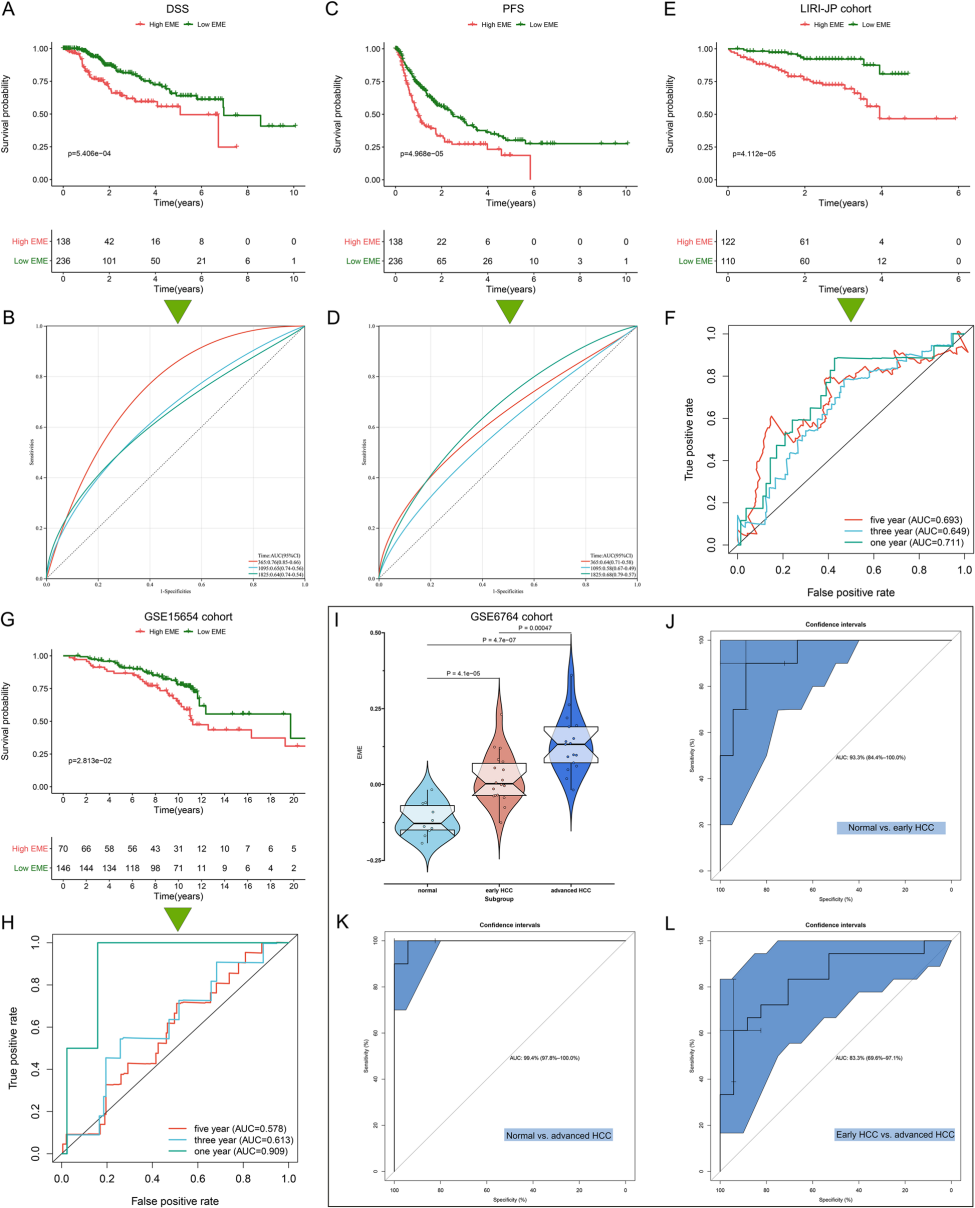
The excellent performance of the EME scoring system in predicting HCC progression. **A** Kaplan–Meier DSS analysis for subgroup patients stratified by the EME. **B** ROC curves of the EME for risk prediction of 1-, 3- and 5-year DSS. **C** Kaplan–Meier PFS analysis for subgroup patients stratified by the EME. **D** ROC curves of the EME for risk prediction of 1-, 3- and 5-year PFS. **E**–**H** Validation of OS outcomes of high and low EME subgroup patients and the prediction performance of the EME by ROC curves in the (**E**, **F**) LIRI-JP and (**G**, **H**) GSE15654 cohorts. **I** Distribution of the EME among normal liver, early-stage HCC, and advanced-stage HCC in the GSE6764 dataset. **J**-**L** ROC curves of the EME for distinguishing normal liver, early-stage HCC, and advanced-stage HCC in the GSE6764 dataset

**Fig. 5:**
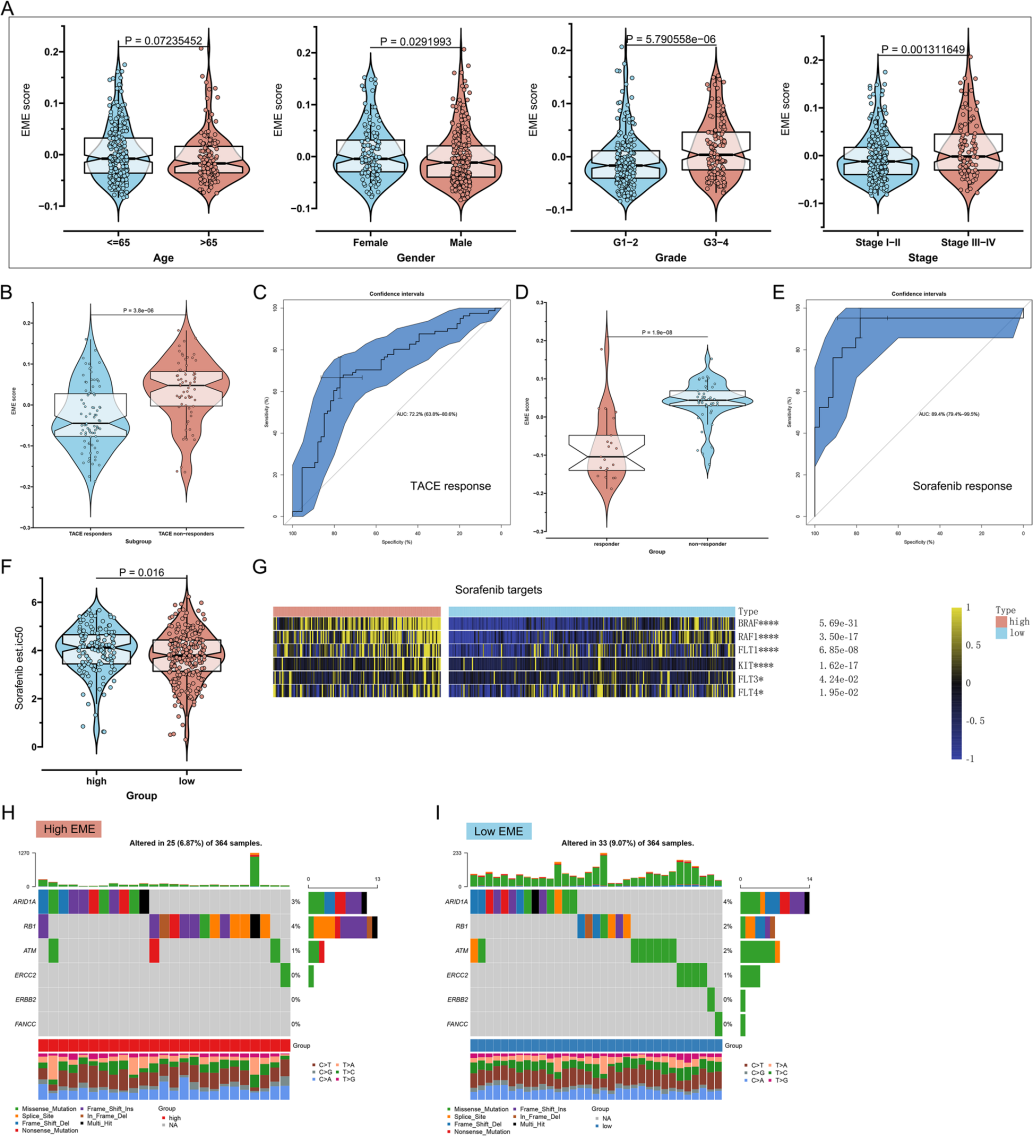
The EME scoring system accurately predicts the responses to mainstream therapies (TACE and sorafenib). **A** Comparison of the EME between subgroup patients stratified by clinicopathological parameters. **B** Difference in the EME between TACE responders and non-responders in the GSE104580 cohort. **C** ROC curves of the EME for evaluating the performance in predicting TACE response. **D** Difference in the EME between sorafenib responders and non-responders in the GSE109211 cohort. **E** ROCs of the EME for assessing the prediction value of the EME in sorafenib response. **F** Difference in the IC50 value of sorafenib between the high- and low-EME patients. **G** Heatmap of the expression of several sorafenib targets in the high- and low-EME patients. **H**, **I** The genomic alterations of sorafenib response-related genes in the high- and low-EME patients. Percentage of mutations is presented on the right panel. Mutation burden is exhibited as a bar plot on the top. Mutation type is displayed in the bottom. *p-value < 0.05; ****p-value < 0.0001

Based on the robust association of the EME with OS, we hypothesized that it would have the same relationships with disease-specific survival (DSS) and PFS. We found that the patients with low EME had a remarkable survival advantage, and the EME enabled to predict the DSS and PFS time (Fig. 4A-D). Aiming to verify the prediction value of the EME, we enrolled the LIRI-JP and GSE15654 independent cohorts. High-EME patients’ OS was remarkably better; additionally, the EME exhibited the excellent predictive power for OS (Fig. 4E-H). The GSE6764 dataset was employed to evaluate whether the EME was associated with the pathological progression of HCC. As the disease progressed, the EME increased gradually (Fig. 4I). ROC curves demonstrated that the EME could accurately differentiate distinct pathological stages of HCC (Fig. 4J-L). Higher EME was found to be linked to advanced pathological grade and stage (Fig. 5A). Altogether, the EME can reflect the progression of HCC.

### The EME scoring system accurately predicts the responses to mainstream therapies (TACE and sorafenib)

In addition to surgical resection, the mainstream therapies against HCC mainly comprise TACE and sorafenib. We evaluated the capacity of the EME scoring system to predict the responses to TACE and sorafenib. In the GSE104580 cohort, TACE non-responders exhibited the higher EME than TACE responders (Fig. 5B). ROC curves were generated to reflect the predictive accuracy. The EME exhibited the high AUC value of 0.722 (Fig. 5C), implying that the scoring system possessed the excellent performance in predicting the response to TACE. Next, we calculated the difference in the EME between sorafenib responders and non-responders in the GSE109211 dataset. As shown in Fig. 5D, we found that the EME score of sorafenib non-responders was higher than that of sorafenib responders. The AUC value was 0.894 (Fig. 5E), demonstrating that the EME had the well accuracy in predicting the response to sorafenib. Further analysis using pRRophetic package showed that the low-EME patients were more sensitive to sorafenib (Fig. 5F). Additionally, we found that several sorafenib targets (BRAF, RAF1, FLT1, KIT, FLT3, FLT4) had the higher expression in the high-EME patients (Fig. 5G). The higher mutation burden of sorafenib response-related genes was found in the low-EME patients (Fig. 5H, I).

### 5mC and m^6^A regulators cooperatively weaken apoptosis and facilitate proliferation for HCC cells

**Fig. 6:**
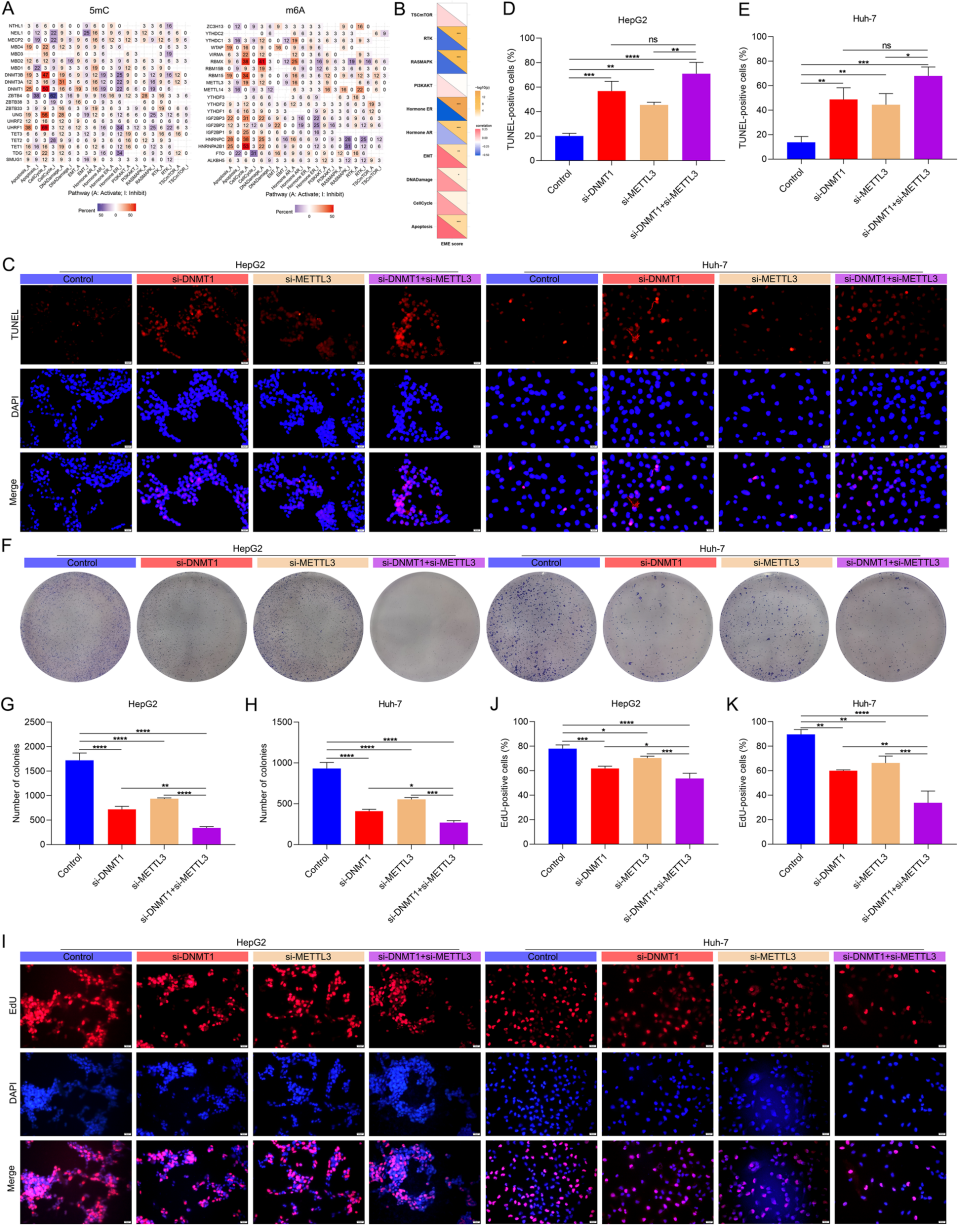
5mC and m^6^A regulators cooperatively weaken apoptosis and facilitate proliferation for HCC cells. **A** Heatmap of the associations between 5mC/m^6^A regulators and the activity of tumorigenic pathways. **B** Heatmap illustrating the correlations of the EME score with the activity of tumorigenic pathways. **C**-**E** TUNEL staining for the apoptosis of HepG2 and Huh-7 cells with si-DNMT1 or/and si-METTL3. Bar, 20 μm. **F**–**H** The number of HCC cell colonies with si-DNMT1 or/and si-METTL3. **I**-**K** EdU staining for the proliferation of HCC cells transfected with si-DNMT1 or/and si-METTL3. Bar, 20 μm. Ns: no significance; *p-value < 0.05; **p-value < 0.01; ***p-value < 0.001; ****p-value < 0.0001

Based on the relevance of the EME scoring system to HCC progression, we next explored the mechanisms by which the interplay between 5mC and m^6^A regulators regulated HCC progression. We found that most 5mC and m^6^A regulators were significantly correlated to tumorigenic pathways (Fig. 6A). Furthermore, the EME score was positively linked with tumorigenic pathways (such as epithelial-to-mesenchymal transition (EMT), DNA damage, and apoptosis; Fig. 6B). Herein, we focused on the synergistic effect of 5mC regulator DNMT1 and m^6^A regulator METTL3 on apoptosis and proliferation of HCC cells. TUNEL staining showed that apoptotic level of HCC cells was enhanced by si-DNMT1 or si-METTL3 in HCC cells (Fig. 6C-E). Simultaneous inhibition of DNMT1 and METTL3 synergistically enhanced apoptosis in HCC cells. We also conducted colony formation assay and EdU staining to evaluate the proliferative capacity of HCC cells. It was found that si-DNMT1 or si-METTL3 mitigated the proliferative capacity of HepG2 and Huh-7 cells, and synergistic effect was observed when DNMT1 and METTL3 were simultaneously inhibited (Fig. 6F-K). Above evidence implied that 5mC and m^6^A regulators cooperatively weakened apoptosis and facilitated proliferation for HCC cells.

### 5mC and m^6^A regulators cooperatively enhance DNA damage repair and cell cycle progression in HCC

**Fig. 7:**
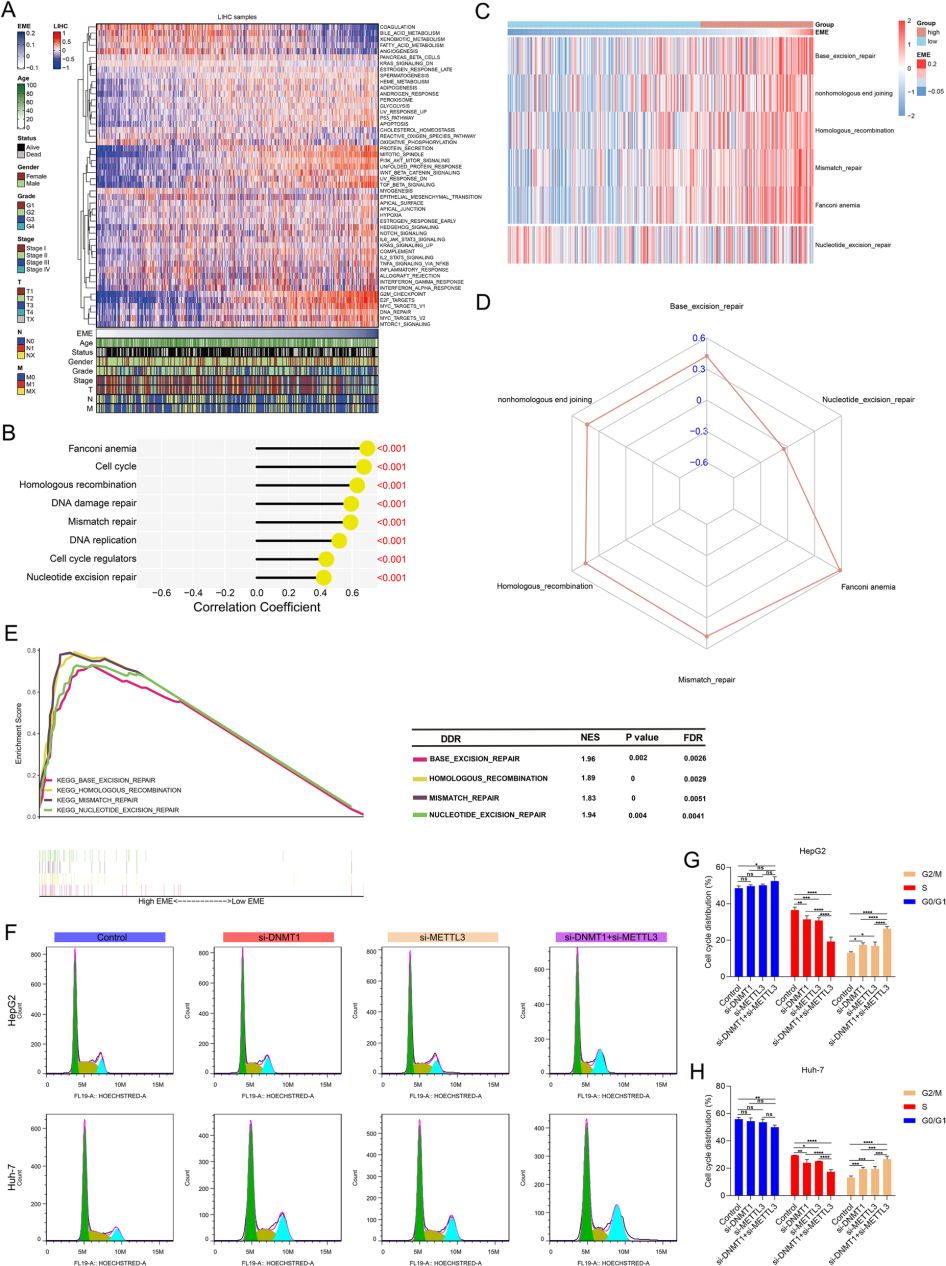
5mC and m^6^A regulators cooperatively enhance DNA damage repair and cell cycle progression in HCC. **A** Heatmap of the activity of the 50 well-defined biological states or processes in the high- and low-EME patients. **B** Associations between the EME and DNA damage repair, and cell cycle pathways. **C** Heatmap of the activity of DNA damage repair pathways in the high- and low-EME patients. **D** Associations between the EME and DNA damage repair pathways. **E** GSEA of DNA damage repair pathways for the high- and low-EME patients. **F**–**H** Flow cytometry for cell cycle distribution of HCC cells with si-DNMT1 or/and si-METTL3. Ns: no significance; *p-value < 0.05; **p-value < 0.01; ***p-value < 0.001; ****p-value < 0.0001

DNA damage response is an important signaling process regulating DNA repair, cell cycle arrest, and maintaining genome homeostasis in response to endogenous and exogenous genotoxic stress. Evidence suggests that HCC cells display a strong DNA repair ability to overcome DNA damage caused by treatment [38]. In Fig. 7A, B, we found that DNA damage repair, cell cycle-related pathways (G2M checkpoint, mitotic spindle, E2F, P53, MYC, PI3K Akt mTOR signaling, TGF-β signaling, etc.) exhibited the strong activation in the high EME patients, implying the cooperative effect of 5mC and m^6^A regulators on DNA damage repair and cell cycle progression. For this reason, we further analyzed the associations between the EME and different DNA damage repair signatures. Base excision repair, nonhomologous end joining, homologous recombination, mismatch repair, and Fanconi anemia were positively correlated to the EME (Fig. 7C, D), and these pathways were significantly enriched in the high EME group (Fig. 7E), implying that the crosstalk between 5mC and m^6^A regulators might be functionally essential for DNA damage response. We examined the effects of knockdown of DNMT1 and METTL3 on cell cycle through flow cytometry. Compared with knockdown of DNMT1 or METTL3 alone, we found that simultaneous inhibition of DNMT1 and METTL3 synergistically induced G2/M cell cycle arrest in HCC cells (Fig. 7F-H).

### 5mC and m^6^A regulators synergistically accelerate migration, invasion and EMT of HCC cells

**Fig. 8:**
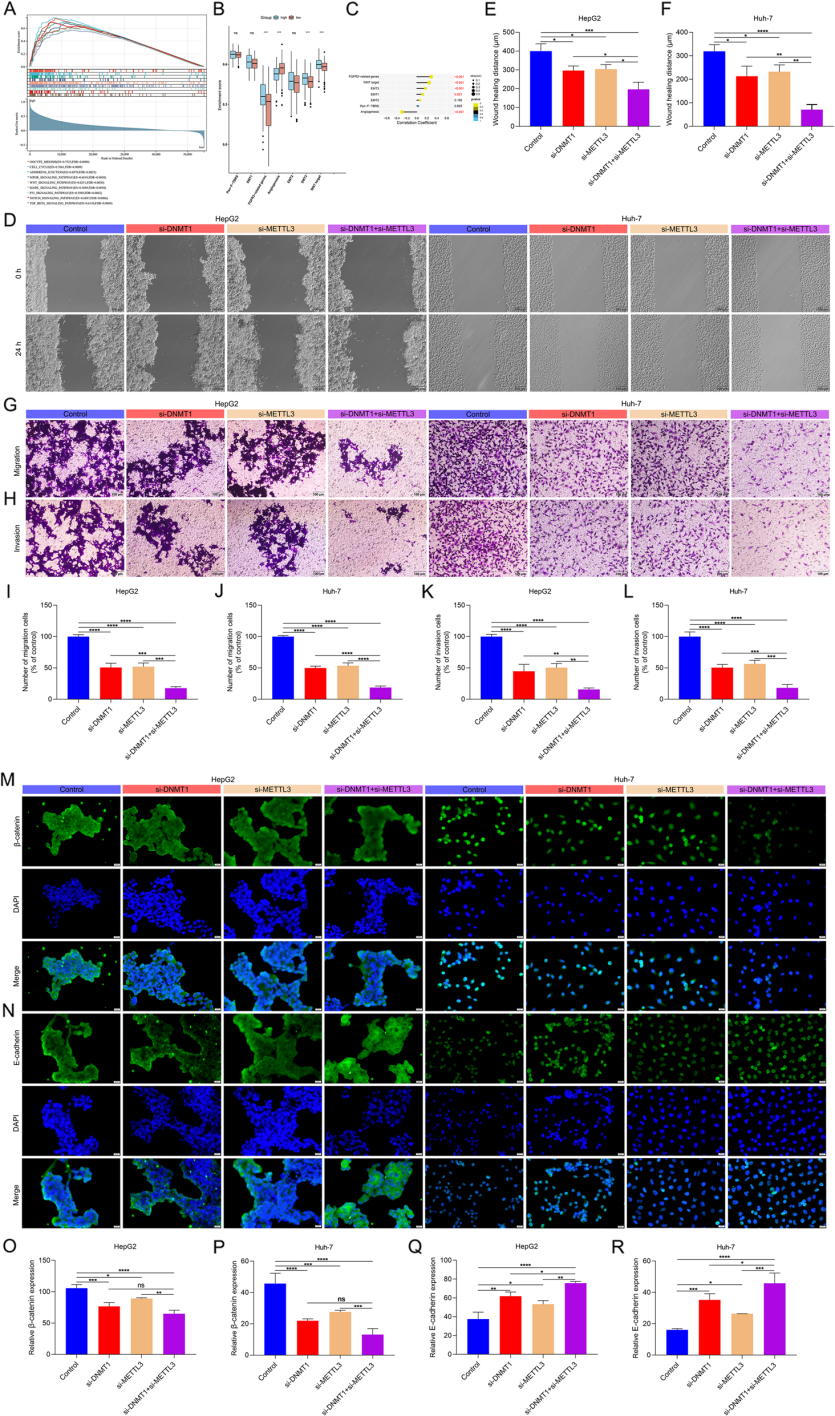
5mC and m^6^A regulators synergistically accelerate HCC cell migration, invasion and EMT. **A** GSEA of KEGG pathways in the low- and high-EME patients. **B** Comparison of the activity of specific EMT-related pathways in the high- and low-EME patients. **C** Associations of the EME with specific EMT-related pathways. **D**-**F** Wound healing of HCC cells with si-DNMT1 or/and si-METTL3. Bar, 200 μm. **G**-**L** Transwell of HCC cells with si-DNMT1 or/and si-METTL3. Bar, 100 μm. **M**-**R** Immunofluorescence of the expression of β-catenin and E-cadherin. Bar, 20 μm. Ns: no significance; *p-value < 0.05; **p-value < 0.01; ***p-value < 0.001; ****p-value < 0.0001

Further analysis showed that the EME was positively linked to EMT process (Fig. 8A-C). Wound healing and transwell assays showed that simultaneous inhibition of DNMT1 and METTL3 synergistically attenuated migration and invasion in HCC cells (Fig. 8D-L). Additionally, simultaneous inhibition of DNMT1 and METTL3 synergistically lowered the expression of β-catenin and enhanced the expression of E-cadherin both in HepG2 and Huh-7 cells (Fig. 8M-R), implying the inactivation of EMT process. In summary, 5mC and m^6^A regulators might synergistically accelerate HCC metastasis.

### Correlation between the EME scoring system and potential extrinsic and intrinsic immune escape mechanisms in HCC

**Fig. 9:**
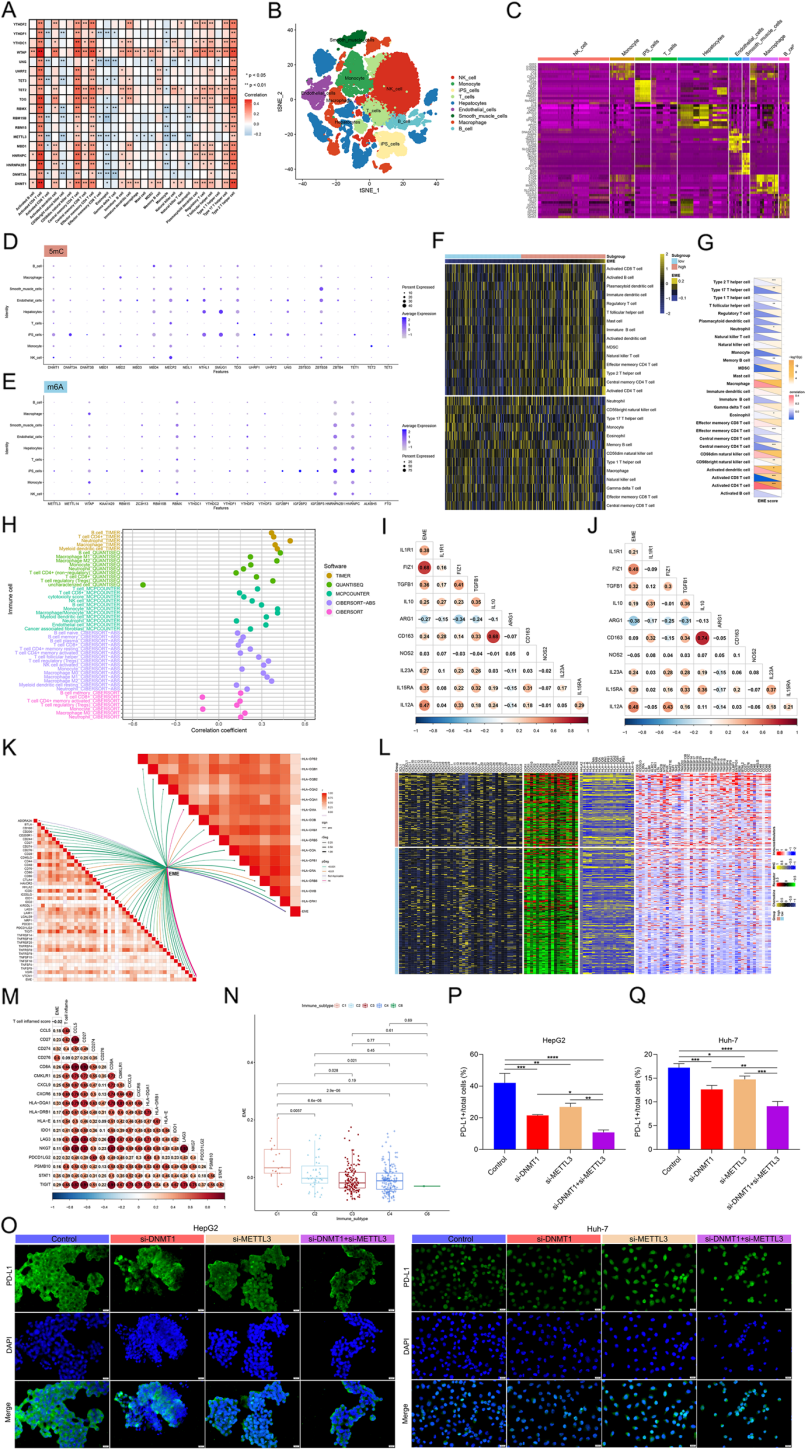
Correlations between the EME scoring system and potential extrinsic and intrinsic immune escape mechanisms in HCC. **A** Heatmap of the relationships between 5mC/m^6^A regulators and tumor-infiltrating immune cells across HCC. **B** t-SNE plot of cell types annotated by unique colors. **C** Heatmap of the expression patterns of the marker genes in each cell type. **D**, **E** Dot-plot showing the expression levels of 5mC and m^6^A regulators in distinct cell types. **F** Heatmap for the infiltration levels of immune cells in the high- and low-EME subsets. **G**, **H** Correlations between the EME and infiltration levels of immune cells through multiple approaches. **I**, **J** Associations between the EME and macrophage markers in TCGA-LIHC and LIRI-JP cohorts. **K** Correlations between the EME and the expression of MHC molecules and immune checkpoints. **L** Differences in the expression of immunomodulators (chemokines, receptors, MHC, and immuno-stimulators) between the high- and low-EME subsets. **M** Associations between the EME and T cell markers. **N** Differences in the EME among known six immune subtypes. **O**-**Q** Immunofluorescence for the expression of PD-L1 in HCC cells with si-DNMT1 or/and si-METTL3. Bar, 20 μm. *p-value < 0.05; **p-value < 0.01; ***p-value < 0.001; ****p-value < 0.0001

Immune surveillance is a key mechanism to prevent tumor development and progression. Extrinsic factors (immune cells) and intrinsic factors (including gene alterations and pathway activity) play important roles in immune escape mechanisms. Hub 5mC/m^6^A regulators were remarkably linked to most tumor-infiltrating immune cells (Fig. 9A). ScRNA-seq can maximize unbiased information to explore transcriptional diversity at the single-cell level. A total of 21 HCC scRNA samples were involved in our study. After quality control, normalization, and dimensionality reduction analysis (Supplementary Fig. 8A-G), the cells were classified as 9 main cell lineages (Fig. 9B), comprising NK cells, monocytes, iPS cells, T cells, hepatocytes, endothelial cells, smooth muscle cells, macrophages, and B cells. Figure 9C visualized the top five markers of the main cell lineages. We found that 5mC/m^6^A regulators were almost expressed in all immune and non-immune cell lineages (Fig. 9D, E). Through implementing different algorithms, we estimated the infiltration levels of immune cells across HCC. As a result, the EME was remarkably linked to the infiltration levels of most immune cell types (Fig. 9F-H), implying the widespread 5mC and m^6^A modifications. Both in TCGA-LIHC and LIRI-JP datasets (Fig. 9I, J), the EME presented positive correlations to macrophage markers (such as FIZ1, TGFB1, IL15RA, and IL12A). In addition, a majority of MHC molecules and immune checkpoints (Fig. 9K) as well as immunomodulators (chemokines, receptors, MHC, and immuno-stimulators; Fig. 9L) were positively correlated to the EME. CD8 + T cell exhaustion is a primary barrier to current immunotherapy [39]. The EME was positively correlated to CD8 + T cell exhaustion markers (CD276, TIGIT, LAG3, CD27, CXCL9, IDO1, etc.; Fig. 9M). Additionally, the remarkable difference in the EME was found among known immune subtypes, with the highest EME in C1 (Fig. 9N). Persistent expression of PD-L1 in tumor cells promotes tumor cells escape from immune surveillance and host T cell exhaustion [40]. Both si-DNMT1 and si-METTL3 mitigated the expression of PD-L1 in HepG2 and Huh-7 cells (Fig. 9O-Q). Simultaneous inhibition of DNMT1 and METTL3 synergistically lowered the expression of PD-L1. In summary, EME scoring system was remarkably linked to extrinsic and intrinsic immune escape mechanisms in HCC.

### The EME scoring system accurately predicts immunotherapeutic response and hyper-progression in HCC

**Fig. 10:**
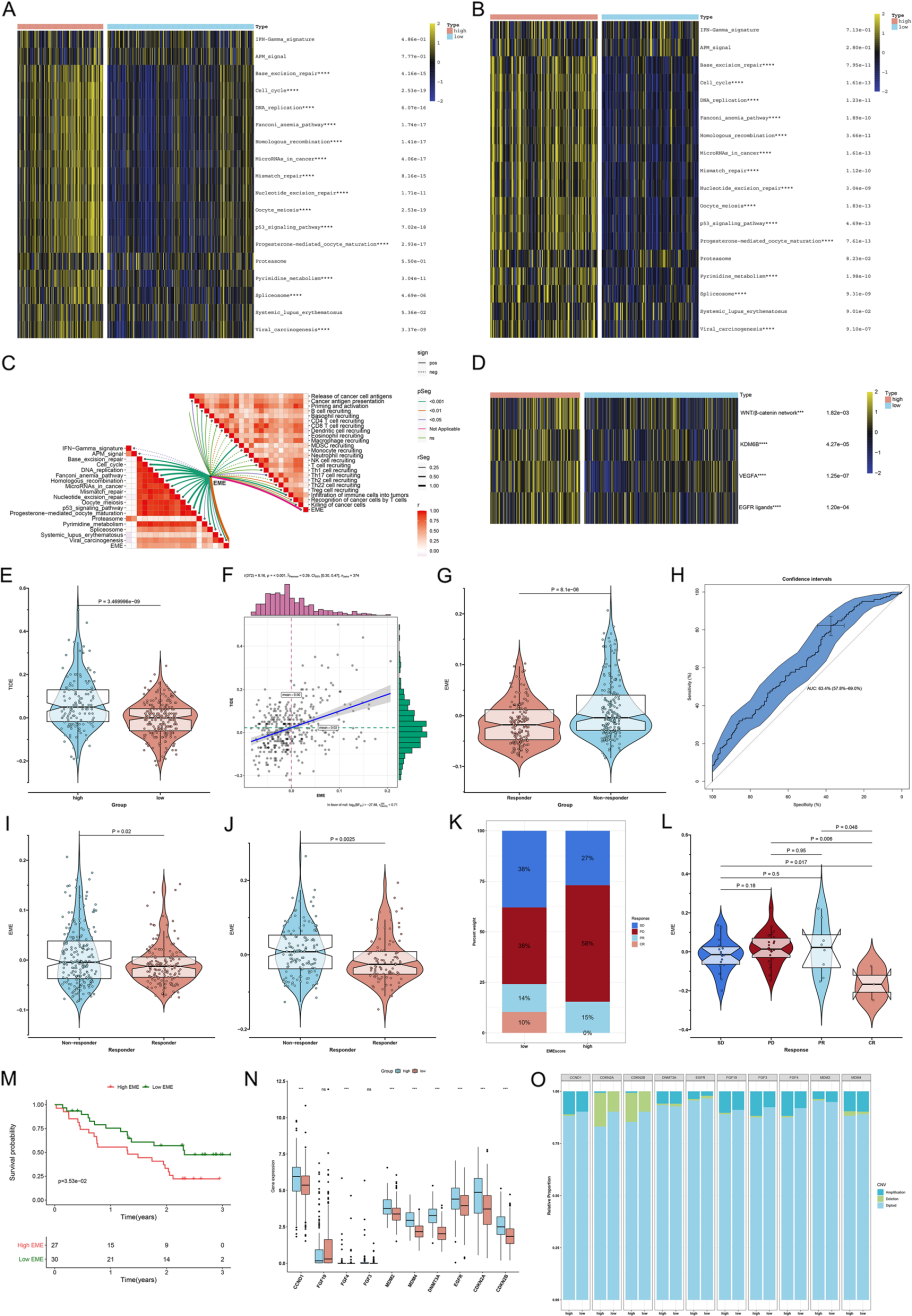
The EME scoring system accurately predicts immunotherapeutic response and hyper-progression in HCC. **A**, **B** Comparison of the enrichment scores of immunotherapy-predicted signatures in the high- and low-EME patients in TCGA-LIHC and LIRI-JP cohorts. **C** Correlations between the EME and immunotherapy-predicted pathways (left panel) and cancer immune cycle (right panel). **D** Comparison of the activity of immune inhibited oncogenic signaling and EGFR ligands in the high- and low-EME subsets. **E** Difference in the TIDE score between the high- and low-EME subsets. **F** Correlation between the EME and TIDE score. **G** Difference in the EME between ImmuCellAI-predicted immunotherapy responders and non-responders. **H** ROC curves for evaluating the predictive accuracy of the EME in immunotherapy response. **I**, **J** Comparison of the EME between TIDE-predicted immunotherapy responders and non-responders in TCGA-LIHC and ICGC cohorts. **K** The proportion of patients in the GSE91061 cohort with different clinical responses (CR, complete response; PR, partial response; SD, stable disease; and PD, progressed disease) in the high- and low-EME subsets. **L** Comparison of the EME among patients with different clinical responses in the GSE91061 cohort. **M** Kaplan–Meier curves of OS between the low- and high-EME patients in the GSE91061 cohort. **N**, **O** Comparison of the mRNA expression and copy number amplification/deletion frequencies of hyper-progression-associated genes in the low- and high-EME patients. Ns: no significance; ***p-value < 0.001; ****p-value < 0.0001

Based on the aforementioned strong relationship between the EME and antitumor immunity, we further evaluated whether the EME could be used to predict the response to immunotherapy. The high-EME subset had the higher enrichment scores of most immunotherapy-predicted gene signatures than the low-EME subset (Fig. 10A), which was confirmed in the LIRI-JP cohort (Fig. 10B). Moreover, we found that the EME exhibited the positive correlations to most immunotherapy-predicted gene signatures (Fig. 10C). The cancer immune cycle includes a series of events required for immune-mediated tumor growth control. Disruption of one or more steps enables tumor cells to escape immune surveillance. The EME was significantly linked to most steps within cancer immune cycle (Fig. 10C). Additionally, the high-EME subset had the higher enrichment scores of immune inhibited oncogenic pathways and EGFR ligands (Fig. 10D). We also computed the TIDE score across HCC, a reliable immunotherapy response predictor. The higher TIDE was observed in the high-EME subset (Fig. 10E), and the TIDE was positively correlated to the EME (Fig. 10F), implying that the low-EME patients were more likely responsive to immunotherapy. ImmuCellAI was also employed to predict cancer immunotherapy response. Immunotherapy non-responders had the higher EME than responders (Fig. 10G). The AUC value was 0.634, implying the well predictive accuracy of the EME in immunotherapy response (Fig. 10H). Immunotherapy response was also predicted by TIDE algorithm. The higher EME was observed in immunotherapy non-responders than responders both in TCGA-LIHC and ICGC cohorts (Fig. 10I, J). The efficacy of the EME in predicting immunotherapy response was also validated in an anti-CTLA4 and anti-PD1 immunotherapy cohort (GSE91061). We computed the EME of patients in this cohort, and assessed the difference in the EME among patients who differently responded to immunotherapy. The low-EME subset presented a higher proportion of complete response (CR) / partial response (PR) compared with the high EME subset (24% vs. 15%; Fig. 10K), and patients who completely responded to anti-CTLA4 or anti-PD1 therapy exhibited the lowest EME (Fig. 10L). Additionally, the low-EME patients had a remarkable survival advantage (Fig. 10M). These results implied that the EME scoring system enabled to reflect the patients’ sensitivity to immunotherapy. We also focused on the immunotherapy-associated hyper-progression. The high-EME subset displayed the higher transcript levels and copy number amplification frequencies of genes positively linked to hyper-progression, such as CCND1, EGFR, and FGF4 (Fig. 10N, O). In summary, the high-EME HCC patients might not benefit from immunotherapy and instead present a higher possibility of hyper-progression.

### Correlation of genomic alterations with the EME in HCC

**Fig. 11:**
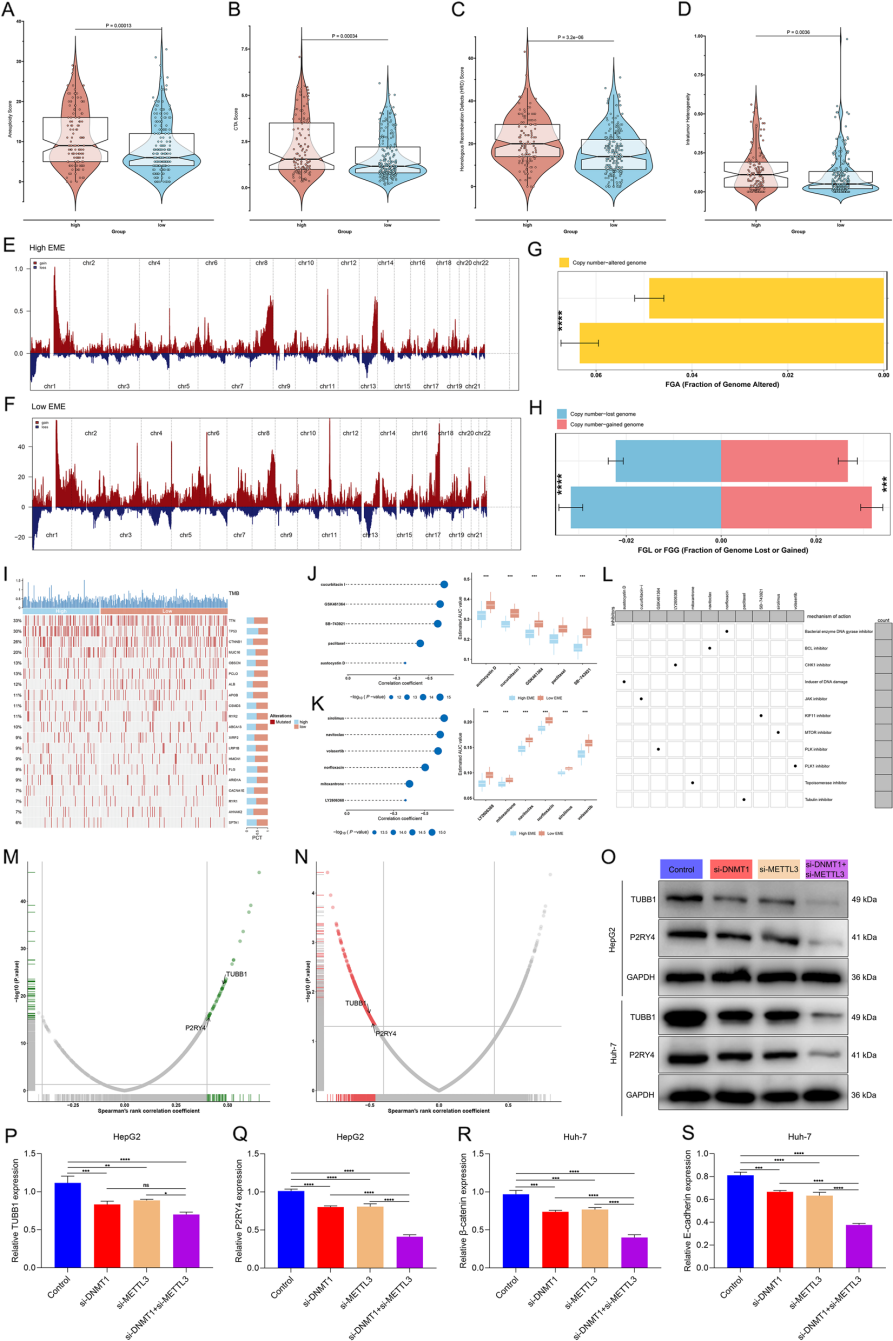
Associations between the EME and genomic alterations, potential therapeutic compounds and druggable targets in HCC. **A**-**D** Comparison of aneuploidy score, CTA score, HRD score, and intratumor heterogeneity in the low- and high-EME patients. **E**, **F** CNVs in the low- and high-EME patients. **G**, **H** Differences in FGA, FGG, and FGL between the high- and low-EME patients. **I** The top twenty mutated genes across HCC patients stratified by the EME. **J**, **K** Spearman correlation analysis on the EME with AUC of CTRP- and PRISM-derived compounds (left), and comparison of AUC between the low- and high-EME subsets (right). **L** Potential working mechanisms of the identified compounds. **M** Spearman’s correlation on the protein expression of compound targets and the EME. Blue dot represents a significant positive correlation (*p*-value < 0.05 and Spearman’s *r* > 0.4). **N** Spearman’s correlation on the CERES score of druggable targets and the EME. Red dot denotes a significant negative correlation (*p*-value < 0.05 and Spearman’s *r* < -0.45). **O**-**S** Western blot for the expression of TUBB1 and P2RY4 in the presence or absence of si-DNMT1 or si-METTL3 in HepG2 and Huh-7 cells. Ns: no significance; *p-value < 0.05; **p-value < 0.01; ***p-value < 0.001; ****p-value < 0.0001

The relationship between the EME and genomic alterations was further analyzed. Firstly, we observed that the high-EME subset had the higher levels of immunogenicity indicators (aneuploidy score, CTA score, HRD score, and intratumor heterogeneity; Fig. 11A-D). Next, we computed the GISTIC score in the high- and low-EME patients. Compared with the high-EME subset, higher GISTIC score and CNV frequency were found in the low-EME subset (Fig. 11E, F). Then, FGA, FGG, and FGL were calculated, respectively. The low-EME subset displayed higher FGA, FGG and FGL levels than the high-EME subset (Fig. 11G, H). This implied that the high 5mC/m^6^A modification might contribute to a reduced copy number gain/loss frequency in HCC. Next, we analyzed the difference in gene mutations between the high- and low-EME subsets. However, we found that there was no remarkable difference between the two subsets (Fig. 11I).

### Identification of potential therapeutic compounds and druggable targets for HCC patients with high EME

The drug response datasets CTRP and PRISM were employed to select potential compounds for the high-EME subset. The compounds with lower AUC in this subset were screened via evaluating the differences in drug response between the high- and low-EME subsets. As a result, five CTRP-derived compounds (paclitaxel, austocystin D, cucurbitacin I, GSK461364, and SB-743921; Fig. 11J) and six PRISM-derived compounds (norfloxacin, LY2606368, mitoxantrone, volasertib, navitoclax, and sirolimus; Fig. 11K) were determined via Spearman correlation analysis between the EME and AUC value (*p*-value < 0.05 and Spearman’s *r* <  − 0.3). In Fig. 11L, we showed the working mechanisms of above compounds: Tubulin inhibitor for paclitaxel; inducer of DNA damage for austocystin D; JAK inhibitor for cucurbitacin I; PLK inhibitor for GSK461364; KIF11 inhibitor for SB-743921; bacterial enzyme DNA gyrase inhibitor for norfloxacin; CHK1 inhibitor for LY2606368; topoisomerase inhibitor for mitoxantrone; PLK1 inhibitor for volasertib; BCL inhibitor for navitoclax; and mTOR inhibitor for sirolimus.

Proteins that were highly positively linked to the EME might exhibit potential therapeutic implications for the high-EME patients. Nevertheless, most human proteins are still undruggable because they are short of distinct active sites to which compounds are capable of binding or residing within cells that are inaccessible to biological agents. Thus, to determine potential druggable targets for the high-EME patients with undesirable survival outcomes, this study obtained the target information of 6,125 compounds. Firstly, we computed Spearman’s correlation of the protein levels of druggable targets with the EME. As a result, 671 druggable targets were determined (*p*-value < 0.05 and Spearman’s *r* > 0.4; Fig. 11M). Then, by implementing correlation analyses on the CERES score of druggable targets and the EME, we determined 497 druggable targets (*p*-value < 0.05 and Spearman’s *r* < -0.45; Fig. 11N). Based on the previously identified druggable targets of HCC [41], two druggable targets including TUBB1 and P2RY4, were finally determined by above analysis. In HCC cells, silencing DNMT1 or METTL3 significantly decreased the expression of TUBB1 and P2RY4 (Fig. 11O-S). Simultaneous suppression of DNMT1 and METTL3 synergistically reduced their expression, implying that suppressing the function of these two targets in the high-EME subset might contribute to beneficial therapeutic response.

## Discussion

The present study unveiled the crosstalk between 5mC-epigenetic mechanism and m^6^A-epitranscriptomic mechanism in HCC at the multiomic levels. The EME scoring system was developed to reflect 5mC/m^6^A modification status in HCC, which may greatly optimize risk stratification, and accurately predict HCC patients’ responses to mainstream therapies (TACE and sorafenib) and immunotherapy as well as hyper-progression. Moreover, the crosstalk between 5mC and m^6^A modifications defined pharmacogenomic landscape including potential therapeutic compounds and druggable targets (TUBB1 and P2RY4). Altogether, our findings laid a solid foundation for epigenetic regulation of HCC as well as paved a new avenue for relevant therapeutic targets.

Interference with tumor growth via 5mC or m^6^A modification levels is a promising therapeutic strategy for HCC. Phenotypic intra-tumor heterogeneity explains the poor efficacy of single-target systemic therapies in HCC [42]. Suppression of 5mC regulator (DNMT1) and m^6^A regulator (METTL3) cooperatively attenuated HCC progression. HCC tumors exhibit intra- and intertumoral heterogeneity at the molecular, histological, and clinical levels. As a multifocal neoplasm, there are differences between distinct lesions in the same patient, known as “inter-lesion” heterogeneity. The complex heterogeneity hinders the development of HCC treatment, notably those who respond differently to drugs and immunotherapies. Hence, stratification of HCC tumors into clinically and molecularly homogeneous subgroups may improve physicians’ therapeutic options. In the present study, the simple and easily applicable EME scoring system was developed to predict clinical outcomes as well as treatment responses (TACE and sorafenib) in patients with HCC, which was verified in the independent and external cohorts.

To better understand the cell cycle, the central molecules that participate in this process have been discovered, along with the introduction of the checkpoint concept [43]. The regulatory network comprising the central molecules is capable of accurately regulating the process. The shared biological feature of HCC is uncontrolled growth, manifested by disordered cell cycle resulting in uncontrollable cell proliferation and attenuated apoptosis [38]. DNA damage repair is a crucial event in mediating cell cycle arrest. The work demonstrated that the EME exhibited positive correlations to diverse DNA damage repair mechanisms, and suppression of DNMT1 and METTL3 synergistically mitigated cell cycle progression of HCC cells.

Complex genomics and tumor microenvironment create a molecular conundrum for the diagnosis and therapy of HCC and result in therapeutic failure and eventually fatal outcomes [44]. A previous study classified liver cancer into four immune subtypes: tumor-associated macrophages, CTNNB1, cytolytic activity (CYT), and regulatory T cells (Tregs) [45]. Among them, CYT and Tregs subtypes are inflamed tumors, and TAMs and CTNNB1 subtypes are non-inflamed tumors. Emerging scRNA-seq can obtain quantitative transcriptomic data at the single-cell level, while eliminating the bias that occur in RNA-seq caused by diverse cell subpopulations [46]. The 5mC and m^6^A regulators were extensively distributed in most immune and non-immune cell populations across HCC. Although HCC tumors harbor significant infiltration of immune cells, they cannot kill tumor cells by inference [47]. Epigenetic and epitranscriptomic modifications can impact the functions and phenotypes of immune cell types for cell killing and functional tuning. The transcriptional and epigenetic landscape of exhausted CD8 + T cells defines exhaustion as a distinct branch of CD8 + T cell differentiation. Selective epigenetic reprogramming alters the T-cell landscape in HCC and may enhance the therapeutic efficacy. T cells cannot infiltrate tumors due to vasculature triggered by tumor cells, chemokines as well as known immunosuppressive cells (MDSCs, immature DCs, macrophages, Tregs, etc.). Epigenetic modulation involves all three aspects. PD-L1 binds to its receptor PD-1, thus leading to immune escape by counteraction of activating signaling on T cells [48]. Moreover, CD80 can bind to PD-L1 and transmit negative signaling. PD-L1 is upregulated in response to some inflammatory signals (IFN-γ, etc.) generated by active T cells during antitumor immune responses [49]. Simultaneous inhibition of DNMT1 and METTL3 synergistically lowered the expression of PD-L1 in HCC cells, thereby suppressing immune escape. Immunosuppression and immune evasion exert key roles in tumorigenesis and tumor progression, in which tumor cells can innately or adaptively express immunosuppressive molecules to escape host immune attack [50]. An in-depth understanding of immune escape mechanisms in tumor cells is critical for overcoming resistance as well as enabling innovative progress in immunotherapy. The advent of ICIs has notably altered the landscape of HCC therapy. Despite these improvements, one of the most relevant unaddressed medical requirements in the field is to identify a biomarker of therapeutic response that may assist determine patients who can respond to ICIs. The EME may predict efficacy of ICI therapy for HCC. From a methodological point of view, the EME scoring system may assist to identify potential responders to immunotherapy and reduce side effects for patients who are not likely to benefit from it.

The work identified promising new pharmaceutical intervention strategies (five CTRP-derived compounds (paclitaxel, austocystin D, cucurbitacin I, GSK461364, and SB-743921) and six PRISM-derived compounds (norfloxacin, LY2606368, mitoxantrone, volasertib, navitoclax, and sirolimus)) for HCC patients with high EME. The newly identified EME may offer an opportunity to test experimental drugs for HCC. Moreover, we determined two druggable targets (TUBB1 and P2RY4) for high-EME patients, which might contribute to beneficial therapeutic response. Nonetheless, the use of the EME scoring system in a clinical setting remains challenging because it requires access to molecular biological platforms for nucleic acid extraction, processing or sequencing. In addition, it depends upon the quality of the samples and is prone to normalization problems. The EME score as possible tool might advance the field of HCC into the precision oncology paradigm. Prospective cohorts are required for optimizing the efficacy of this available therapeutic response tool as well as solving other remaining issues. Altogether, our study might assist to determine whether the EME score can be used in clinical practice, thus assisting therapeutic decisions as well as selecting the patient subgroup with the highest clinical benefits from TACE, sorafenib or ICIs.

## Conclusion

Altogether, our work unveils that the crosstalk between 5mC-epigenetic and m^6^A-epitranscriptomic mechanisms may contribute to understanding the mechanisms that underlie HCC pathogenesis and progression. The EME scoring system that reflects 5mC and m^6^A modification levels can accurately predict clinical outcomes and therapeutic response (TACE, sorafenib, and immunotherapy) for HCC patients, and thus assists clinicians to design a personalized treatment plan. Additionally, we determine potential therapeutic compounds and druggable targets (TUBB1 and P2RY4) for the high-EME patients with poor prognosis. Altogether, our study extends the knowledge of the crosstalk between 5mC and m^6^A modifications, and provides novel strategies for treating HCC.

## Supplementary Information

**Additional file 1: Supplementary figure 1.** Landscape of the expression levels and genetic alterations of 5mC/m^6^A regulators across 33 cancer types. (A) Gene expression profiling of 5mC/m^6^A regulators across pan-cancer. For a given 5mC/m^6^A regulator in each cancer type, the median expression value is shown. (B, C) Spearman correlations of CNVs with transcript levels of 5mC/m^6^A regulators in pan-cancer, reflecting the gene expression significantly affected by CNVs. (D, E) Pie chart for heterozygous and homozygous CNVs of 5mC/m^6^A regulators across cancer types.

**Additional file 2: Supplementary figure 2.** Landscape of the methylation levels of 5mC/m^6^A regulators across pan-cancer. (A, B) Correlations of methylation and mRNA expression of 5mC/m^6^A regulators. (C, D) Differential methylation of 5mC/m^6^A regulators between tumor and paired normal tissues in each cancer type. (E, F) Survival differences between high and low methylation of 5mC/m^6^A regulators in each cancer type, reflecting survival affected by methylation.

**Additional file 3: Supplementary figure 3.** Drug sensitivity of 5mC/m^6^A regulators in pan-cancer. (A, B) Spearman correlation analysis between the expression levels of 5mC/m^6^A regulators and the small molecule compound sensitivity (IC50).

**Additional file 4: Supplementary figure 4.** Co-expression of TET3 and YTHDC1 in HCC cells. (A-F) Western blot for the expression of TET3 and YTHDC1 in the presence or absence of si-TET3 or si-YTHDC1 in HepG2 and Huh-7 cells. **p-value<0.01; ***p-value<0.001; ****p-value<0.0001.

**Additional file 5: Supplementary figure 5.** Prognostic value of 5mC/m^6^A regulators. (A) Heatmap showing the univariate cox regression analysis of 5mC/m^6^A regulators with OS across pan-cancer. (B) Forest plot for the univariate cox regression analysis of 5mC/m^6^A regulators with HCC patients’ OS.

**Additional file 6: Supplementary figure 6.** Functional enrichment analysis of hub 5mC/m^6^A regulators. (A) Biological process; (B) cellular component; (C) molecular function; (D) KEGG pathways.

**Additional file 7: Supplementary figure 7.** Subgroup analysis of HCC patients stratified by distinct clinicopathological factors. Kaplan-Meier curves of OS between high and low EME HCC patients in each subgroup of (A, B) age ≥65 and <65, (C, D) female and male, (E, F) G1-2 and G3-4, (G, H) stage I-II and stage III-IV, (I, J) T1-2 and T3-4, and (K, L) HBV infection and non-infection.

**Additional file 8: Supplementary figure 8.** Quality control, normalization, and dimensionality reduction analysis of HCC scRNA-seq from the GSE149614 cohort. (A) The number of genes, and summary expression values of all genes in single cells, and the percentage of mitochondrial genes. (B) Removal of single cells with the high percentage of mitochondrial genes and doublets. (C) Identification of highly variable genes. (D-F) Scaling the scRNA-seq. (G) Identification of the number of PCs with JackStraw.

## Data Availability

All data generated or analysed during this study are included in this published article [and its supplementary information files].

## Abbreviations

HCC: Hepatocellular carcinoma
HBV: Hepatitis B virus
HCV: Hepatitis C virus
BCLC: Barcelona Clinic Liver Cancer
TACE: Transarterial chemoembolization
PFS: Progression-free survival
OS: Overall survival
DSS: Disease-specific survival
5mC: 5-Methylcytosine
m^6^A: N^6^-methyladenosine
EME: Epitranscriptomic module eigengene
TCGA: The Cancer Genome Atlas
RSEM: Root square error method
TPM: Transcripts per kilobase million
RMA: Robust multiarray averaging
MAF: Mutation Annotation Format
FGA: Fraction of genome alteration
FGG: Fraction of genome gained
FGL: Fraction of genome lost
IFN-γ: Interferon gamma
TGF-β: Transforming growth factor beta
CTA: Cancer testis antigen
HRD: Homologous recombination deficiency
CNV: Copy number variation
GDSC: Genomics of Drug Sensitivity in Cancer
CTRP: Cancer Therapeutics Response Portal
PPI: Protein–protein interaction
WGCNA: Weighted gene co-expression network analysis
GO: Gene Ontology
KEGG: Kyoto Encyclopedia of Genes and Genomes
GSVA: Gene set variation analysis
GSEA: Gene Set Enrichment Analysis
AUC: Area under the curve
ssGSEA: Single-sample gene set enrichment analysis
ImmuCellAI: Immune Cell Abundance Identifier
TIDE: T cell dysfunction and exclusion
IC50: Half-maximal inhibitory concentration
TUNEL: Terminal-deoxynucleoitidyl transferase mediated nick end labeling
scRNA-seq: Single-cell sequencing
PCs: Principal components
t-SNE: T-distributed stochastic neighbor embedding
CCLs: Cancer cell lines
CCLE: Cancer Cell Line Encyclopedia
ANOVA: Analysis of variance
HRs: Hazard ratios
ROC: Receiver operating characteristic
PCA: Principal component analysis
