# Supplementary figures and images for: Crosstalk between 5-methylcytosine and N6-methyladenosine machinery defines disease progression, therapeutic response and pharmacogenomic landscape in hepatocellular carcinoma
Source: Mol Cancer. 2023 Jan 10;22:5. doi: 10.1186/s12943-022-01706-6 (PMC9830866; doi:10.1186/s12943-022-01706-6)

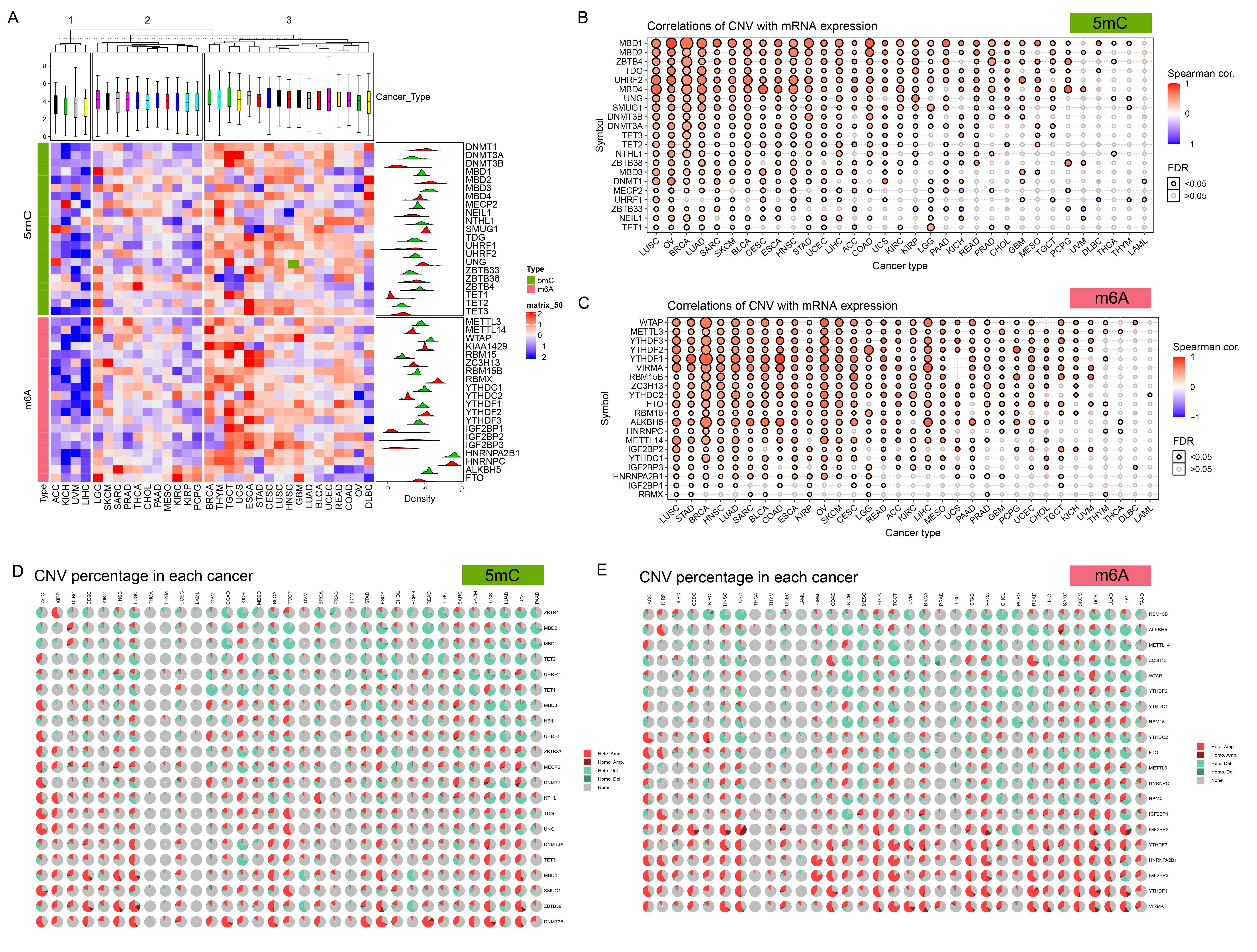

Supplement: Supplementary file 1 — Additional file 1: Supplementary figure 1. Landscape of the expression levels and genetic alterations of 5mC/m6A regulators across 33 cancer types. (A) Gene expression profiling of 5mC/m6A regulators across pan-cancer. For a given 5mC/m6A regulator in each cancer type, the median expression value is shown. (B, C) Spearman correlations of CNVs with transcript levels of 5mC/m6A regulators in pan-cancer, reflecting the gene expression significantly affected by CNVs. (D, E) Pie chart for heterozygous and homozygous CNVs of 5mC/m6A regulators across cancer types. [file 12943_2022_1706_MOESM1_ESM.tif]

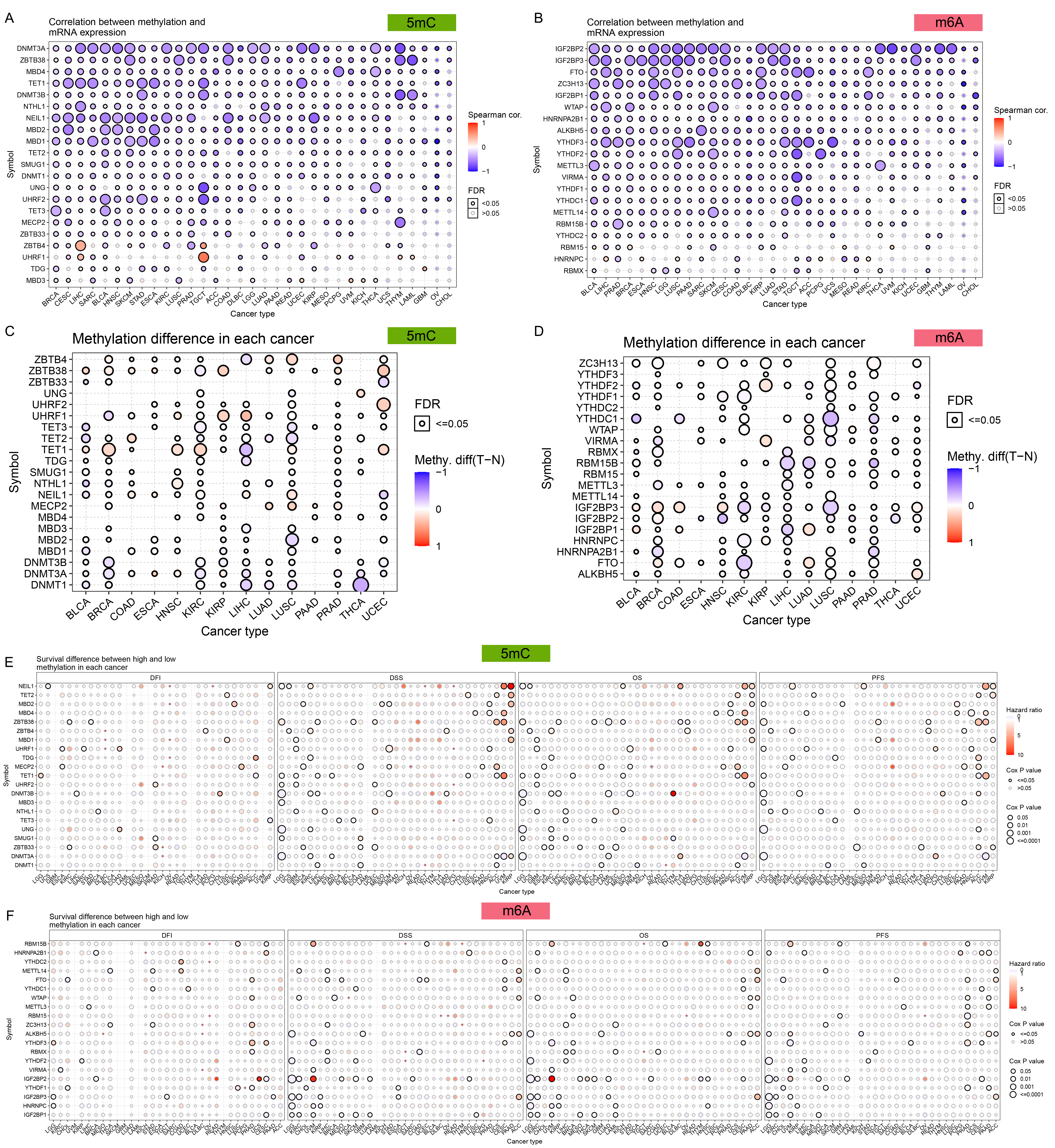

Supplement: Supplementary file 2 — Additional file 2: Supplementary figure 2. Landscape of the methylation levels of 5mC/m6A regulators across pan-cancer. (A, B) Correlations of methylation and mRNA expression of 5mC/m6A regulators. (C, D) Differential methylation of 5mC/m6A regulators between tumor and paired normal tissues in each cancer type. (E, F) Survival differences between high and low methylation of 5mC/m6A regulators in each cancer type, reflecting survival affected by methylation. [file 12943_2022_1706_MOESM2_ESM.tif]

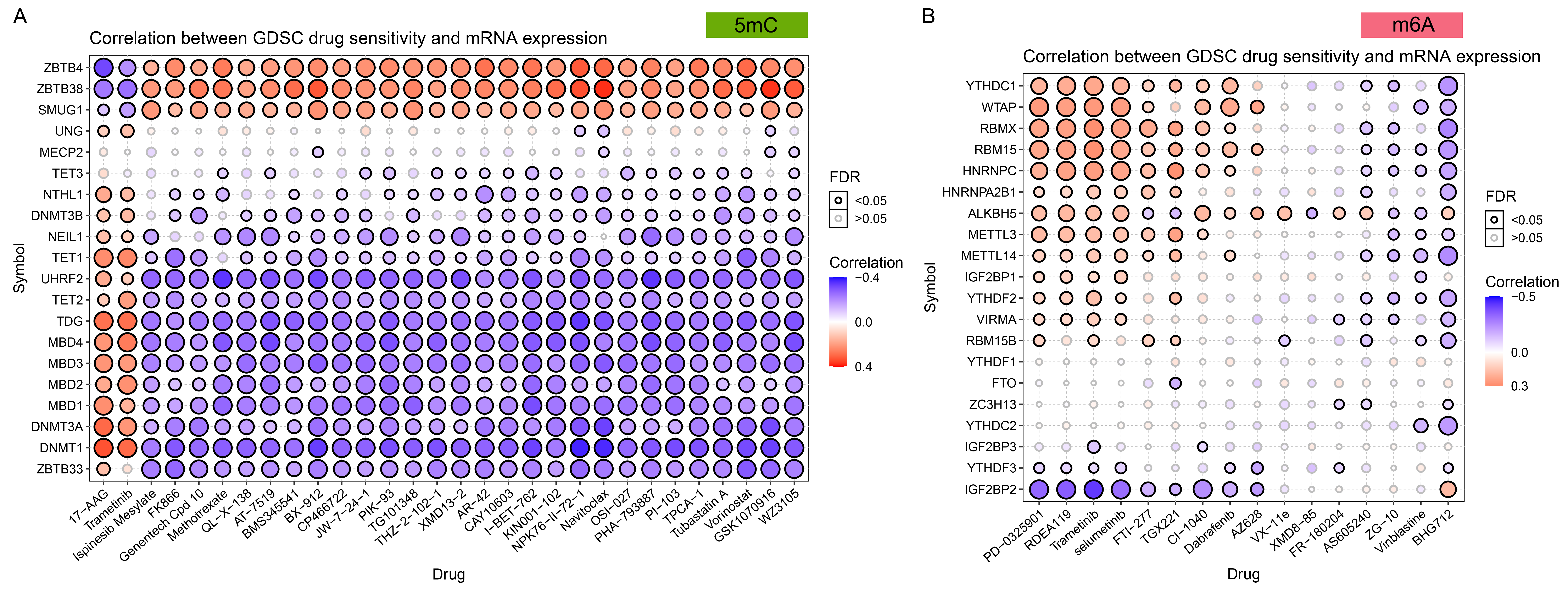

Supplement: Supplementary file 3 — Additional file 3: Supplementary figure 3. Drug sensitivity of 5mC/m6A regulators in pan-cancer. (A, B) Spearman correlation analysis between the expression levels of 5mC/m6A regulators and the small molecule compound sensitivity (IC50). [file 12943_2022_1706_MOESM3_ESM.tif]

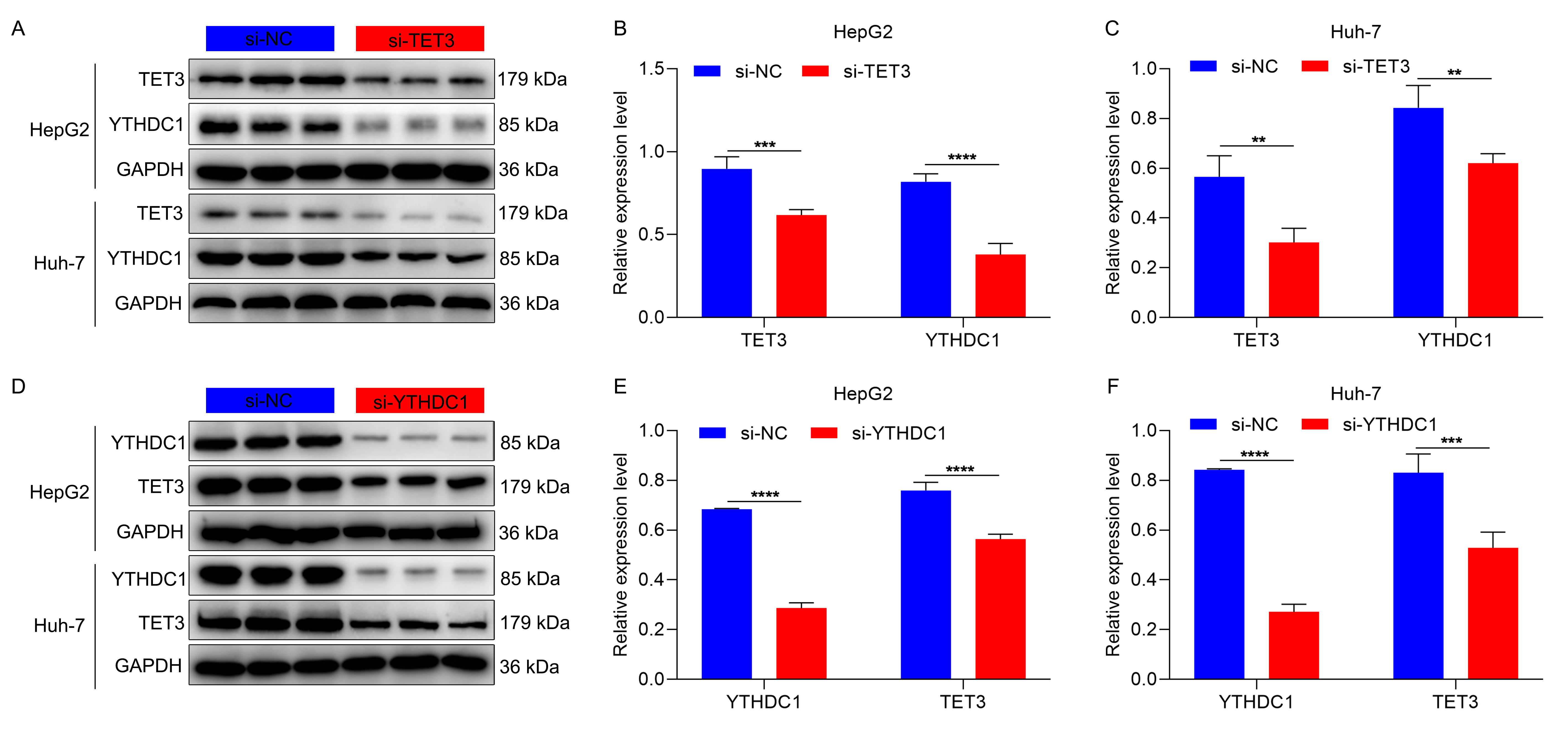

Supplement: Supplementary file 4 — Additional file 4: Supplementary figure 4. Co-expression of TET3 and YTHDC1 in HCC cells. (A-F) Western blot for the expression of TET3 and YTHDC1 in the presence or absence of si-TET3 or si-YTHDC1 in HepG2 and Huh-7 cells. **p-value<0.01; ***p-value<0.001; ****p-value<0.0001. [file 12943_2022_1706_MOESM4_ESM.tif]

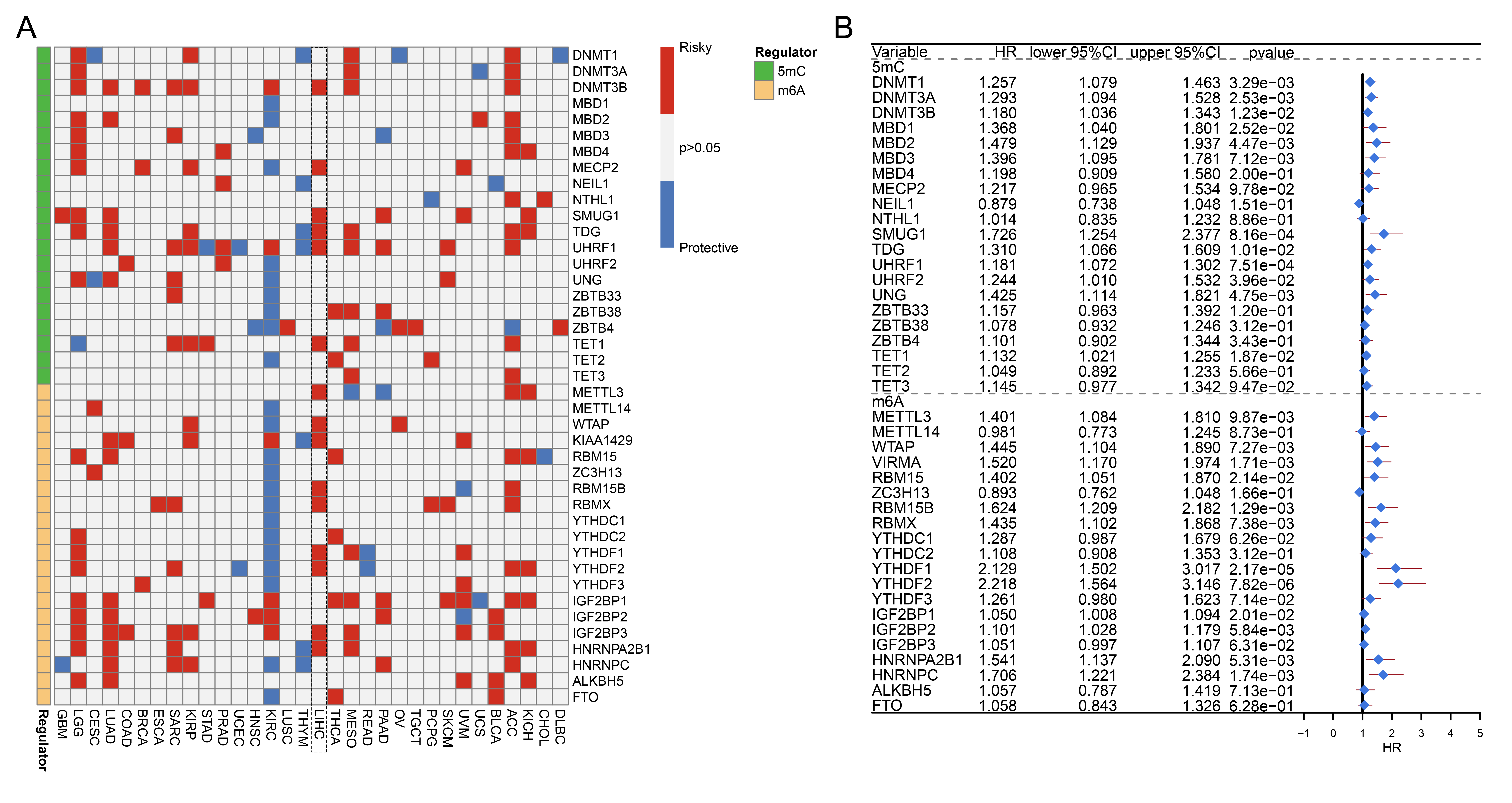

Supplement: Supplementary file 5 — Additional file 5: Supplementary figure 5. Prognostic value of 5mC/m6A regulators. (A) Heatmap showing the univariate cox regression analysis of 5mC/m6A regulators with OS across pan-cancer. (B) Forest plot for the univariate cox regression analysis of 5mC/m6A regulators with HCC patients’ OS. [file 12943_2022_1706_MOESM5_ESM.tif]

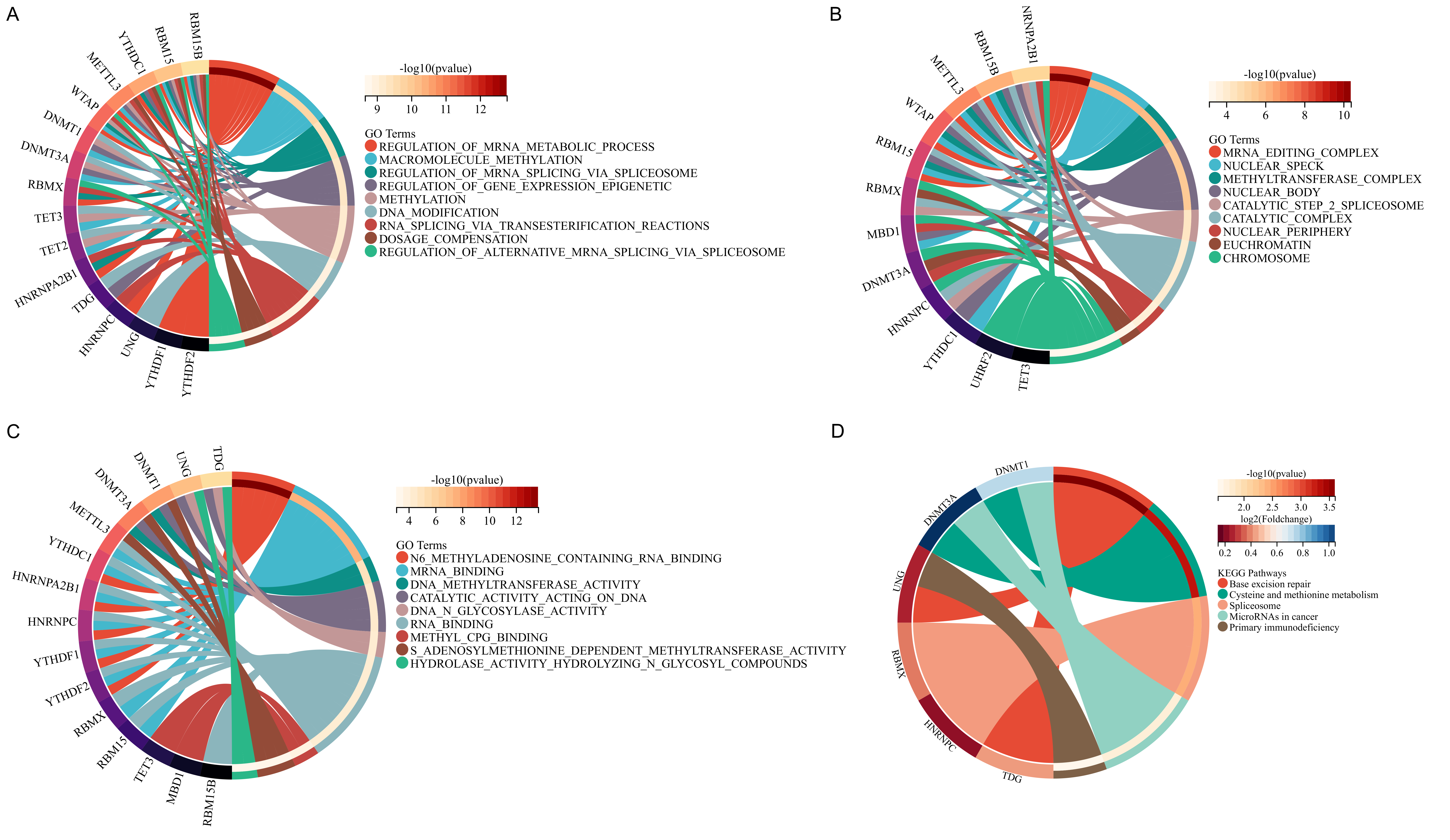

Supplement: Supplementary file 6 — Additional file 6: Supplementary figure 6. Functional enrichment analysis of hub 5mC/m6A regulators. (A) Biological process; (B) cellular component; (C) molecular function; (D) KEGG pathways. [file 12943_2022_1706_MOESM6_ESM.tif]

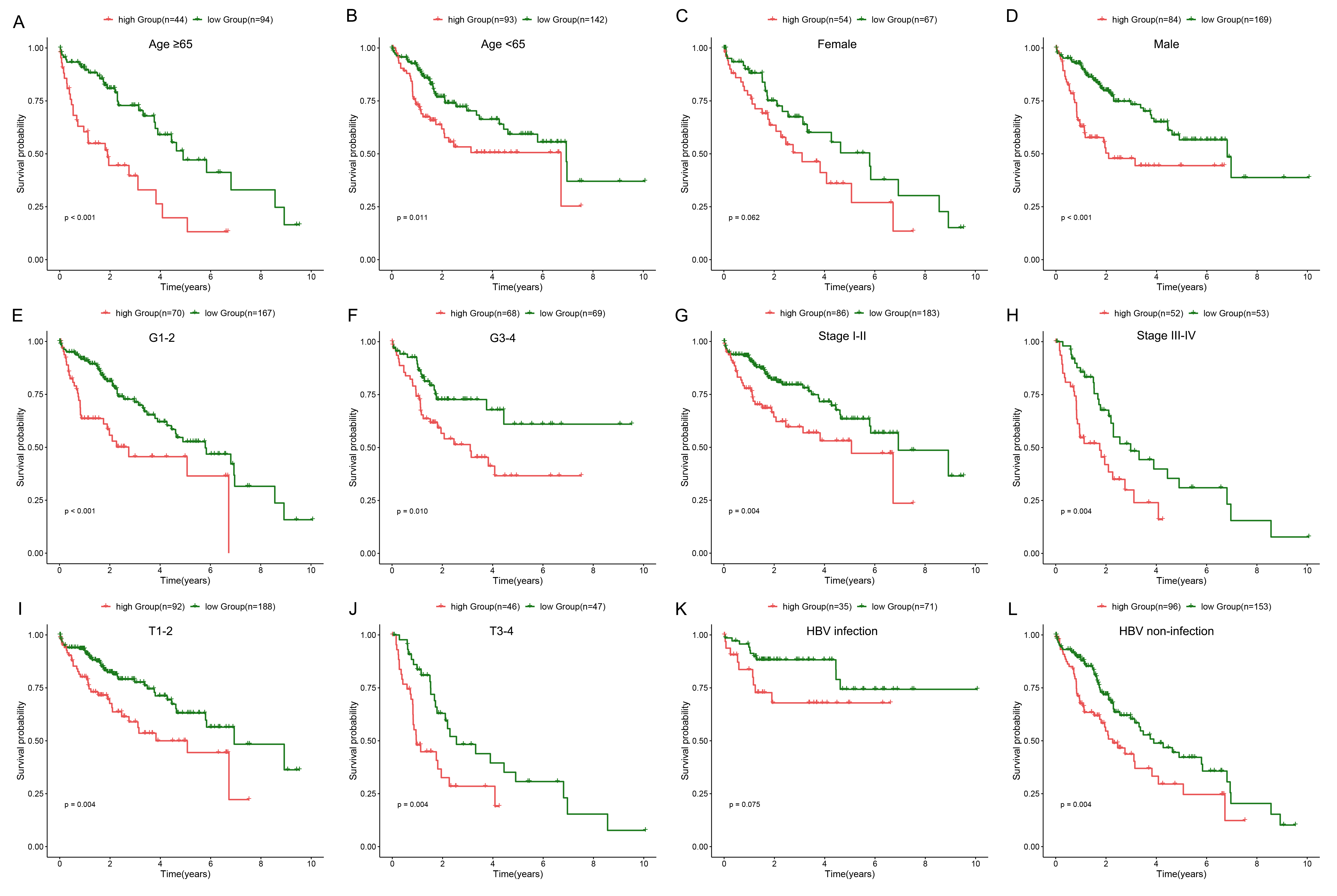

Supplement: Supplementary file 7 — Additional file 7: Supplementary figure 7. Subgroup analysis of HCC patients stratified by distinct clinicopathological factors. Kaplan-Meier curves of OS between high and low EME HCC patients in each subgroup of (A, B) age ≥65 and <65, (C, D) female and male, (E, F) G1-2 and G3-4, (G, H) stage I-II and stage III-IV, (I, J) T1-2 and T3-4, and (K, L) HBV infection and non-infection. [file 12943_2022_1706_MOESM7_ESM.tif]
